# Ferroptosis and cuproptosis in head and neck squamous cell carcinoma: interconnected mechanisms and therapeutic implications

**DOI:** 10.3389/fphar.2026.1694895

**Published:** 2026-02-13

**Authors:** Yinan Liu, Yanru Li

**Affiliations:** 1 Department of Otorhinolaryngology Head and Neck Surgery, Beijing Tongren Hospital, Capital Medical University, Beijing, China; 2 Key Laboratory of Otorhinolaryngology Head and Neck Surgery (Capital Medical University), Ministry of Education, Beijing, China

**Keywords:** cuproptosis, ferroptosis, HNSCC, mechanism, therapy

## Abstract

Head and Neck Squamous Cell Carcinoma (HNSCC) is a highly prevalent malignant neoplasm worldwide. Iron and copper metabolism disorder regulate ferroptosis and cuproptosis, two forms of cell death, respectively, and play key roles in the progression and treatment response of HNSCC. Recent studies have shown that these two death pathways have complex interactions, which together affect the malignant progression and tolerance of HNSCC, providing potential targets for its treatment. This review systematically elucidates the interconnected regulatory networks linking ferroptosis and cuproptosis in HNSCC, with particular emphasis on the clinical significance of associated biomarkers for diagnosis and therapy. We further discuss the potential advantages of dual-targeting strategies and critically evaluate current challenges and limitations in translational applications. By providing novel insights into metal ion-dependent cell death mechanisms, this review establishes a theoretical foundation for developing innovative combinatorial therapeutic approaches against HNSCC.

## 1 Introduction

Head and Neck Squamous Cell Carcinoma (HNSCC) as the most common malignant tumor in the head and neck region is a prevalent cancer worldwide, originating from the mucosal epithelium of the oral cavity, pharynx, and larynx (Johnson et al., 2020). Ferroptosis is an iron-mediated form of regulated cell death characterized by excessive lipid peroxidation, which has important pathological significance in various cancers, neurodegenerative diseases, and bears a close association with the progression and therapeutic management of HNSCC (Dixon et al., 2012; Chung et al., 2023). Cuproptosis is a form of copper-dependent cell death induced by excessive copper ions, which triggers proteotoxic stress through copper-binding proteins in the mitochondrial respiratory chain, leading to cell death. Copper binds to multiple key molecules in tumor cells, activating typical signaling pathways such as MAPK and PI3K/AKT, thereby participating in the regulation of cell proliferation, metastasis, and drug resistance (Xie et al., 2023b). The latest research shows that there is a cross - regulation mechanism between ferroptosis and cuproptosis through pathways such as ROS accumulation, mitochondrial damage, and TCA cycle, suggesting that they may coordinately regulate the fate of tumors (Liu and Chen, 2024). To this end, this study therefore endeavors to investigate this cross-talk in the context of HNSCC, based on a comprehensive literature search of PubMed and Web of Science (2000–2025) using relevant keywords, with priority given to highly cited and preclinical studies.

## 2 Mechanisms and crosstalk between ferroptosis and cuproptosis in HNSCC

### 2.1 Metal ion homeostasis disorder

There is a significant imbalance in iron homeostasis in HNSCC. Compared with normal tissues, the iron content and the expression levels of FTH and ferritin light chain (FTL) are increased in HNSCC tissues. Especially in metastatic HNSCC, and the upregulation of FTH1 is an independent prognostic factor (Hu et al., 2018; 2021). FTH1 knockout induces ferroptosis in oral squamous cell carcinoma (OSCC) cells, thereby inhibiting the proliferation, EMT, and invasive phenotype of tumor cells (Wen et al., 2024). In addition, the high expression of TFR1 is associated with a poor prognosis in patients with HNSCC (Liu F. et al., 2022). Emerging evidence demonstrates that dysregulated ACSL4 expression and activity significantly play a role in promoting pathological progression of HNSCC, particularly through modulating tumor invasiveness, metabolic reprogramming, and regulated cell death mechanisms. In esophageal squamous cell carcinoma (ESCC), ACSL4 can serve as a valuable prognostic biomarker for long-term survival (Wang X. et al., 2024). In OSCC patients, receptor accessory protein 6 (REEP6) is overexpressed, which induces ER stress through ACSL4 and mediates ferroptosis, leading to the progression of OSCC (Xia L. et al., 2024). Podoplanin-positive cancer-associated fibroblasts (PDPN + CAFs) enhance OSCC invasiveness by suppressing ferroptosis through the FTX/FEN1/ACSL4 signaling axis (Li et al., 2023d). Emerging evidence implicates multiple microRNAs in the modulation of iron homeostasis in HNSCC. For example,miR-107, miR-148a, and miR-210 can inhibit HNSCC ferroptosis by regulating TFR (Datta et al., 2012; Yoshioka et al., 2012; Gee et al., 2014). Meanwhile, miR-17-92 cluster confers ferroptosis resistance through ACSL4 downregulation (Xiao et al., 2019).

There is a strict mutual regulatory relationship between intracellular copper and iron levels: copper deficiency impairs iron absorption, reduces the iron-binding ability of transferrin, and ultimately leads to erythropoietic disorders and anemia (Gao et al., 2021). Dysregulation of copper ion homeostasis also exists in HNSCC. In HNSCC tissues, the expression of copper transport protein ATP7B is upregulated, and cisplatin resistance is generated by promoting copper efflux (Yoshizawa et al., 2007). A detailed description of the regulatory mechanisms for intracellular iron and copper ions is provided in Supplementary Material 1.

### 2.2 Mitochondrial metabolism

**FIGURE 1:**
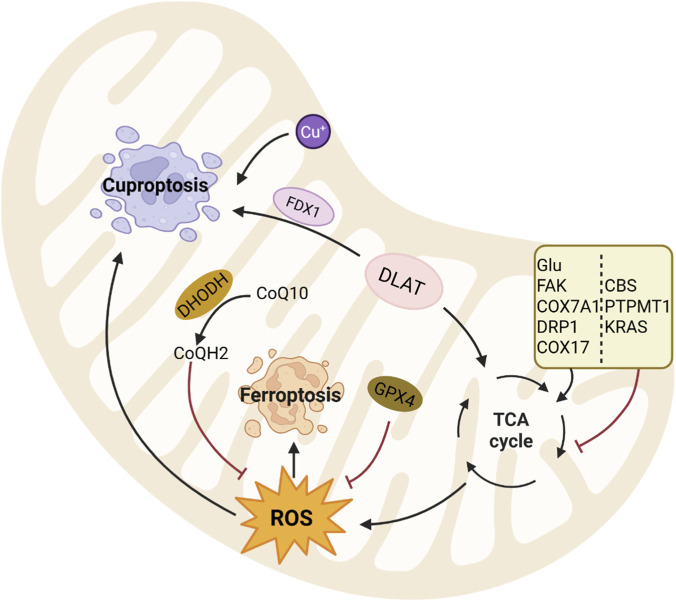
Schematic illustration of crosstalk between ferroptosis and cuproptosis within mitochondria. (Refer to Supplementary Material 1 for mechanistic details. → denotes activation; ⊥ indicates inhibition).

Mitochondria are the core hub for the regulation of ferroptosis and cuproptosis, and the activities of key enzymes all converge in the tricarboxylic acid cycle (TAC): the key enzyme DLAT acylation modification mediated by ferredoxin 1 (FDX1) and lipoic acid synthase (LIAS) enables copper to bind and trigger cuproptosis, while the TAC mainly drives ferroptosis through ROS generation (Wang et al., 2020; Chung et al., 2023). Meanwhile, dihydroorotate dehydrogenase (DHODH) is an inner mitochondrial membrane enzyme that suppresses ferroptosis through its generation of ubiquinol, which acts as a lipid-soluble antioxidant to neutralize lipid peroxyl radicals (Mao et al., 2021; Zhang et al., 2022a). A schematic overview of this intricate crosstalk between ferroptosis and cuproptosis within mitochondria is presented in Figure 1. And the distinct mitochondrial mechanisms of ferroptosis and cuproptosis in HNSCC are detailed in Supplementary Material 1.

### 2.3 GSH metabolism

**FIGURE 2:**
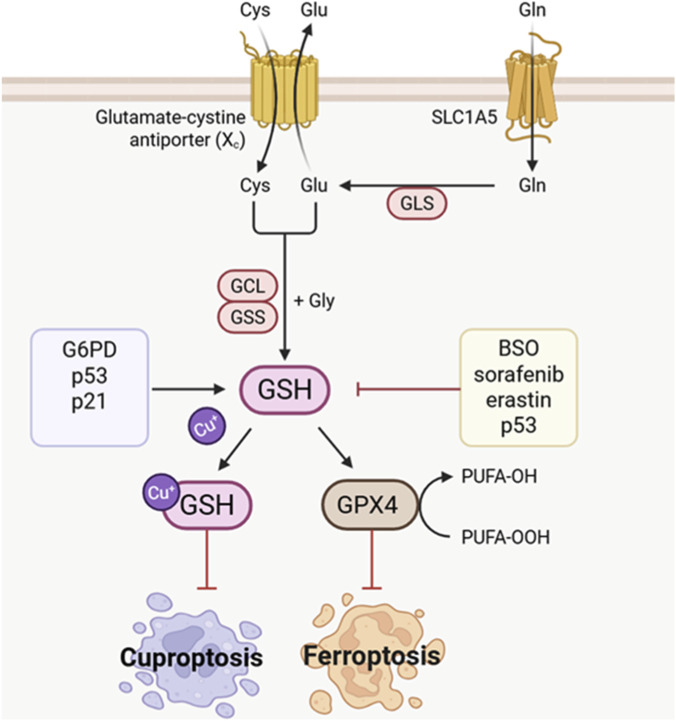
Schematic illustration of GSH modulating ferroptosis and cuproptosis. (Refer to Supplementary Material 1 for mechanistic details. → denotes activation; ⊥ indicates inhibition).

GSH, a tripeptide antioxidant comprising glutamic acid, cysteine, and glycine, serves as the primary intracellular reducing agent in biological systems. Through its sulfhydryl group (-SH), GSH participates in critical physiological processes including redox homeostasis maintenance, free radical scavenging, and detoxification metabolism (Liu T. et al., 2022). As illustrated in Figure 2, beyond functioning as an essential cofactor for GPX4 activity to suppress lipid peroxidation, GSH also chelates free copper ions, thereby mitigating DLAT acylation-induced protein aggregation (Liu and Chen, 2024). Consequently, GSH exerts dual protective effects against both ferroptosis and cuproptosis. SLC7A11 takes up cystine to synthesize GSH, and both SLC7A11 and the GSH/GPX4 axis demonstrate elevated expression levels during the progression of HNSCC cell lines (Shi et al., 2021). The specific mechanisms underlying the crosstalk among ferroptosis, cuproptosis, and GSH in HNSCC are detailed in Supplementary Material 1.

### 2.4 Autophagy

**FIGURE 3:**
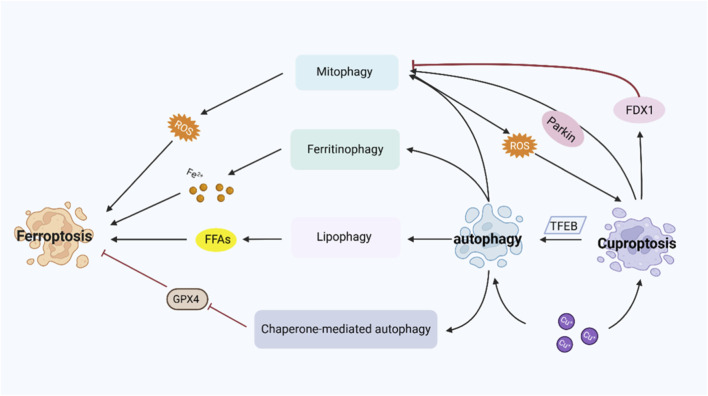
Schematic diagram of autophagy involved in the regulation of ferroptosis and cuproptosis. (Refer to Supplementary Material 1 for mechanistic details. → denotes activation; ⊥ indicates inhibition).

Autophagy is a biological process driven by autophagy-related proteins (ATG) and their partners. It enables the intracellular degradation and recycling of damaged or redundant organelles and proteins, mainly through autophagosome formation and lysosomal degradation, serving dual roles in both physiological maintenance and pathological adaptation (Levine and Kroemer, 2019; Antonelli et al., 2024). The cytotoxic effects of copper and iron ions are primarily mediated by the production of ROS through Fenton and Fenton-like reactions. Besides directly inducing cell death, ROS can also activate the autophagy pathway to enhance cellular defense. However, excessive activation of autophagy may form a positive feedback loop by selectively degrading key protective factors such as antioxidant proteins, thus accelerating the process of cell death (Denton and Kumar, 2019). As summarized in Figure 3, autophagy serves as a pivotal regulatory mechanism that intersects with both ferroptosis and cuproptosis, either by degrading key factors (e.g., ferritin, GPX4) or through complex bidirectional signaling with copper-mediated cell death. In HNSCC, autophagy and ferroptosis interweave through key molecules such as NCOA4, GPX4, and USP14, jointly influencing tumor progression, therapeutic resistance, and prognosis (Song J. et al., 2024; Tao et al., 2024). Although there are currently few direct studies on cuproptosis and autophagy in HNSCC, in many other cancer types, it has been found that copper homeostasis closely links cuproptosis with autophagy through key molecules such as FDX1 and TFEB, and copper ion-induced autophagic degradation of GPX4 further links this interaction with ferroptosis. For details on the underlying mechanisms, please refer to Supplementary Material 1.

### 2.5 Important signaling pathways and molecules

#### 2.5.1 p53

**FIGURE 4:**
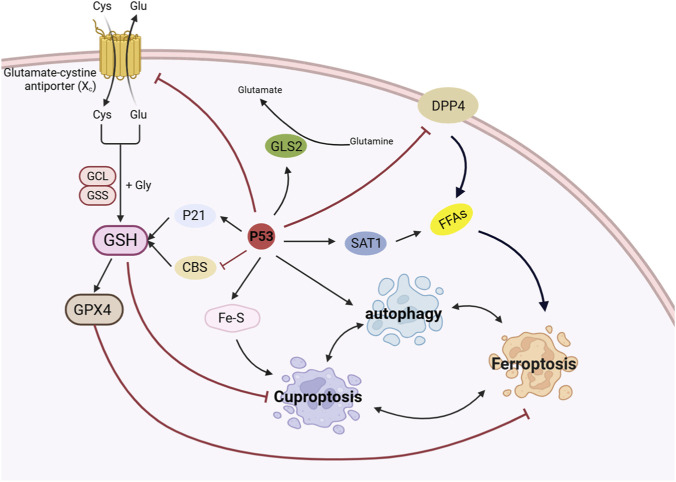
Role of p53 pathway in ferroptosis and cuproptosis. (Refer to Supplementary Material 2 for mechanistic details. → denotes activation; ⊥ indicates inhibition).

The tumor suppressor p53 serves as a central regulator of intracellular ion homeostasis, maintaining metal ion balance through a dual mechanism: preventing excessive accumulation of labile iron/copper ions under physiological conditions, while eliminating damaged cells via ferroptosis and cuproptosis pathways under pathological conditions. As comprehensively illustrated in Figure 4, under amino acid deprivation, p53 upregulates GLS2 expression, thereby enhancing ROS generation and lipid metabolism. p53 enhances lipid peroxidation by promoting the expression of SAT1, while simultaneously inhibiting DPP4-dependent lipid peroxidation. p53 is also involved in Fe-S cluster assembly to affect cuproptosis. p53 exerts biphasic control over glutathione metabolism in cell death regulation. On one hand, it attenuates GSH biosynthesis through CBS and SLC7A11 suppression, thereby potentiating both ferroptotic and cuproptotic cell death. Conversely, activation of the p53-p21 axis elevates GSH levels, augmenting GPX4-mediated clearance of lipid peroxides. Furthermore, p53 regulates autophagy to take part in determining cell fate, thereby forming an intricate metabolic-death regulatory network.

In HNSCC, multiple molecules promote the progression of ferroptosis by regulating the p53 signaling pathway. For instance, compared with normal esophageal tissues, the overexpression of transgelin (TAGLN) in patients with ESCC promotes ferroptosis through its interaction with p53 (Chen et al., 2023). p53 interacts with miR-34c to transcriptionally regulate xCT expression, thereby modulating cellular susceptibility to ferroptosis in HNSCC (Gupta et al., 2025). Research in OSCC has identified that attenuated ferroptosis facilitates cancer cell proliferation and tumorigenesis, potentially mediated through p53 downregulation and subsequent activation of the SREBP1-GPX4 axis (Fukuda et al., 2021). Clinical studies have demonstrated that recombinant human p53 adenovirus (rAd-p53) significantly enhances radiosensitivity in recurrent NPC patients (Ma et al., 2017). However, there is currently a lack of direct evidence of the association between cuproptosis and p53 in HNSCC, and future studies should further elucidate the molecular details of p53 regulatory networks to facilitate the development of p53 homeostasis-based precision therapeutics.

#### 2.5.2 NRF2

Nuclear factor E2-related factor 2 (NRF2), a stress-responsive transcription factor, maintains cellular redox homeostasis through multi-dimensional regulatory mechanisms. Its downstream target gene network is extensively involved in establishing and maintaining the antioxidant defense system (Zhang et al., 2017). In HNSCC, NRF2 activation promotes ferroptosis resistance by upregulating key molecules such as SLC7A11, GPX4, FTL/FTH1, and FPN. Conversely, NRF2 knockout reduces GSH levels thus enhances ferroptotic sensitivity (Chen et al., 2009; Chung et al., 2023). Although there is no direct research on cuproptosis and NRF2 in HNSCC currently, a growing body of research has demonstrated the important role of NRF2 in regulating cuproptosis recently in other cancers, with its mechanisms involving the regulation of ion metabolism, intermediate metabolism, GSH metabolism, and antioxidant stress responses (Tang D. et al., 2024). NRF2 activation counteracts copper overload and suppresses cuproptosis mainly by upregulating key effectors including metallothioneins (MT-1/MT-2), glutathione synthesis enzymes (GCLM, GCLC), and the copper exporter ATP7B (Gudekar et al., 2020; Du et al., 2024; Liu et al., 2024). A comprehensive summary of the specific mechanisms is provided in Supplementary Material 2.

**FIGURE 5:**
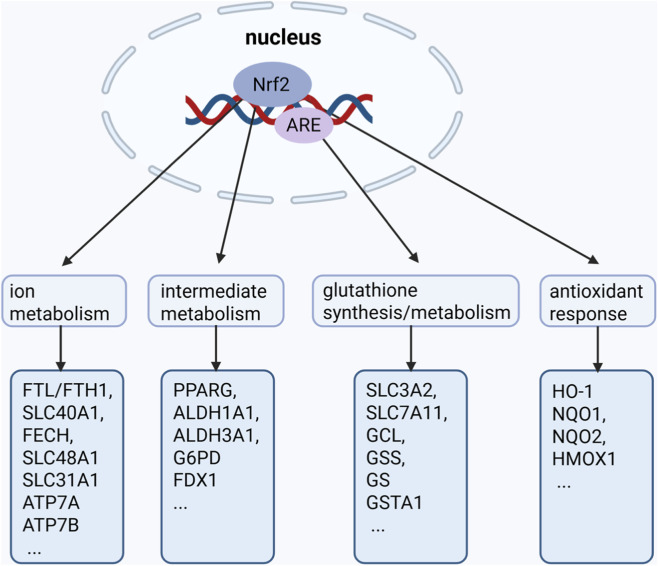
The function of Nrf2 signaling in neutralizing ferroptosis and cuproptosis. (Refer to Supplementary Material 2 for mechanistic details).

Nrf2 confers resistance to metal ion-dependent cell death through transactivation of downstream target genes as outlined in Figure 5, making targeted inhibition of NRF2 to simultaneously induce ferroptosis and cuproptosis in cancer cells a potential new strategy for HNSCC treatment.

#### 2.5.3 PI3K/AKT

In addition to p53 and NRF2, several other molecules and signaling pathways co-regulate ferroptosis and cuproptosis in tumors, with the PI3K-AKT-mTOR signaling pathway being particularly important. Research has established that persistent activation of the PI3K-AKT-mTOR cascade markedly increases tumor cell resistance to both oxidative stress and ferroptosis. This cytoprotective effect is mediated through the SREBP1/SCD1 axis, which promotes lipid biosynthesis and membrane stabilization (Yi et al., 2020). Maternal embryonic leucine zipper kinase (MELK) increases the expression level of the cuproptosis-related signature (CRS) gene DLAT by activating the PI3K/mTOR pathway, stabilizes mitochondrial function, and ultimately promotes HCC progression. Elesclomol, however, can abrogate these changes, indicating the regulatory role of the PI3K/mTOR pathway in ferroptosis and cuproptosis, particularly in cuproptosis (Li Z. et al., 2023). Michael et al. found that HNSCC cells harboring genetic alterations in the PIK3CA/PTEN pathway display significant sensitivity to glutamine metabolism inhibition, suggesting that hyperactive mTOR signaling may enhance their dependence on glutamine metabolism (Allevato et al., 2024). Fibroblast growth factor 6 (FGF6) may activate the PI3K/AKT/MAPK signaling pathway through specific binding to the FGFR4 receptor, thereby regulating the expression of apoptosis-related proteins and proliferation factors. Activation of this signaling pathway may contribute to the malignant progression of OSCC by inhibiting ferroptosis (Zhang et al., 2024c). Currently, although systematic research on the role of the PI3K-AKT pathway in the combined therapeutic strategies of ferroptosis and cuproptosis for HNSCC remains limited, its therapeutic potential in this field warrants attention. Hyperactivation of PI3K-AKT drives metabolic reprogramming and redox imbalance, which represent key vulnerabilities targeted by ferroptosis and cuproptosis. Therefore, targeted inhibition of PI3K-AKT may synergistically enhance the efficacy of iron/copper-dependent cell death inducers. Future studies should explore combination strategies involving PI3K inhibitors and ferroptosis/cuproptosis inducers. By leveraging the metabolic remodeling effects mediated by this pathway, new insights may be provided for HNSCC treatment.

It is noteworthy that the current understanding of p53, NRF2 and PI3K-AKT pathway regulation in ferroptosis and cuproptosis is largely derived from pan-cancer analyses, which may obscure the unique regulatory landscape in HNSCC. To advance precision therapeutics for HNSCC, future studies are urgently needed to delineate the precise regulatory maps of ferroptosis and cuproptosis specific to this malignancy, with a particular focus on elucidating the mechanisms of cuproptosis.

## 3 The prognostic significance of genes associated with ferroptosis and cuproptosis in HNSCC

### 3.1 Ferroptosis-related genes of HNSCC

Ferroptosis has been recognized as a potential targeted strategy for HNSCC treatment (Yang M. et al., 2023). Accumulating evidence demonstrates that ferroptosis-related genes (FRGs) exhibit significant prognostic value, potentially serving as novel molecular biomarkers for clinical outcome prediction in HNSCC patients. These findings highlight the crucial value of FRGs in prognostic evaluation, overall survival prediction, and guiding treatment decisions for HNSCC. Prognostic models for HNSCC constructed based on FRGs have demonstrated favorable predictive efficacy, providing an important basis for formulating clinical diagnosis and treatment regimens.

Li et al. constructed a ferroptosis-related prognostic model consisting of 5 genes (SLC7A11, AURKA, TRIB3, AKR1C3, and Cav1), which achieved good predictive performance (Li C. et al., 2021). Aurora kinase A (AURKA) is an important cell cycle regulator involved in mitotic spindle formation, chromosomal segregation, and cytokinesis, with well-established oncogenic properties across multiple cancer types (Du R. et al., 2021). Emerging evidence now identifies AURKA as a novel modulator of ferroptosis in malignant cells (Jia et al., 2024). The regulation of AURKA can exert significant impacts on multiple signaling pathways in tumors, such as the PI3K/Akt, mTOR, β-catenin/Wnt, and NF-κB pathways (Du R. et al., 2021). AURKA demonstrates significant overexpression in HNSCC tissues, and AURKA knockdown suppresses ESCC progression through ferroptosis induction (Mi et al., 2024). Furthermore, it has been demonstrated that HNSCC patients with TP53 mutations and HPV exhibit upregulated AURKA expression, which is associated with poor prognosis and cisplatin resistance in HNSCC patients. Therapeutic targeting of AURKA using either the specific inhibitor Alisertib or genetic knockdown approaches significantly attenuates the proliferative and migratory capacities of HNSCC cells (Jia et al., 2024). Another study showed that AURKA regulates apoptosis and epithelial-mesenchymal transition in OSCC by mediating the production of ROS (Dawei et al., 2018). An intricate negative feedback loop exists between TRIB3 and activating transcription factor 4 (ATF4). TRIB3 not only functions as a target gene of ATF4 but also inhibits AKT1 phosphorylation, thereby reducing ATF4 expression. ATF4 influences ferroptosis through multiple pathways. Several of its target genes, including HSPA5, SLC7A11, and NUPR1, act as negative regulators of ferroptosis. Conversely, other ATF4 target genes such as BTG1, DDIT3, CHAC1, and SLC1A5 positively regulate ferroptosis (Tang H. et al., 2024). Consequently, the role of TRIB3 in ferroptosis is rather complex. In current research, TRIB3 primarily exerts a tumor-promoting effect on HNSCC by inhibiting ferroptosis (Chen L. et al., 2024). The aldo-keto reductase (AKR) family comprises enzymes that mitigate the cytotoxic potential of aldehydes and ketones through their conversion into corresponding alcohols. Therefore, ferroptosis resistance may be associated with the detoxification activity of AKR, as this activity leads to a reduction in lipid peroxidation products, whose accumulation is a key process in ferroptosis (Gagliardi et al., 2019). Caveolin-1 (Cav1) serves as an essential scaffolding protein that modulates both cellular signaling pathways and vesicular trafficking processes. Cav1-associated signaling can regulate lipid metabolism and induce cell death. Both Cav1 and Cav2 are commonly upregulated in HNSCC and predict poor prognosis (He J. et al., 2023). Overexpression of Cav1 reduces the expression of NOX1 and ACSL4 while increasing the expression of FTH1 and GPX4, thereby promoting ROS production and decreasing tumor cell sensitivity to ferroptosis. However, when Cav-1 is knocked down in HNSCC, ferroptosis is activated, and cell proliferation, invasion, and migration are all inhibited (Lu T. et al., 2022; Tang Z. et al., 2024). Meanwhile, Cav-1 plays an unexpected role in stabilizing ATP7A protein expression by preventing its ubiquitination and proteasomal degradation. Additionally, it increases SOD3 activity in endothelial cells, thereby preventing vascular oxidative stress-mediated endothelial dysfunction (Sudhahar et al., 2020).

Huang et al. systematically evaluated the correlation between FGSs and the prognosis and tumor microenvironment (TME) of HNSCC patients, and constructed clustering subgroups and an FGS model. All 28 prognostic differentially expressed genes (DEGs) in the model were highly expressed in tumor tissues, including ATG5, AURKA, CAV, FTH1, SLC3A2, SLC7A11, and SOCS1(Huang Z. et al., 2022). The study found that CD276 was significantly associated with FGS risk scores and poor survival outcomes. As an immune checkpoint molecule, CD276 inhibits T-cell activation and proliferation while promoting tumor invasion and metastasis. It is highly expressed in most fibroblasts and associated with poor prognosis (Huang Z. et al., 2022), suggesting its potential as a therapeutic target for HNSCC patients with poor prognosis. ATG5, an autophagy-related gene, also acts as a key driver of ferroptosis (Hou et al., 2016). ATG5 is a key gene in FGS, which is highly expressed in high-risk patients, and a negative correlation exists between ATG5 and CD8^+^ T cells (Huang Z. et al., 2022). The suppressor of cytokine signaling 1 (SOCS1) can downregulate the expression of SLC7A11 and GSH by modulating the expression of p53 target genes, thereby enhancing the sensitivity of tumor cells to ferroptosis (Saint-Germain et al., 2017).

In the OSCC10-FRDEGs risk scoring model developed by Tang et al., CA9, Cav1, AURKA, and EGFR serve as the hub genes of the model and act as markers and molecular targets for poor prognosis in OSCC patients (Tang Z. et al., 2024). Among them, carbonic anhydrase (CA) plays a role in balancing hypoxia, iron metabolism, and redox regulation in tumor cells. CA9 expression is significantly upregulated in OSCC samples. Knockdown of CA9 can inhibit the proliferation, invasion, and metastasis of HNSCC cells by promoting ferroptosis. Ferroptosis inhibitors can counteract the effects induced by such knockdown (Li et al., 2019). Over 90% of HNSCC cases overexpress epidermal growth factor receptor (EGFR). Studies have found that the combined application of the ferroptosis inducer RSL3 and the EGFR monoclonal antibody cetuximab can significantly inhibit the survival of nasopharyngeal carcinoma cells (CNE-2) that are insensitive to ferroptosis induction alone. Moreover, blocking EREG/GPX4 sensitizes head and neck cancer to cetuximab through the induction of ferroptosis (Liu S. et al., 2022; Jehl et al., 2023). In addition, another study has shown that EGFR regulates the malignant mechanisms of OSCC by modulating the autophagy-related protein sequestosome-1 (Tseng et al., 2021).

Numerous ubiquitination-related enzymes have been demonstrated to be associated with ferroptosis in HNSCC cells. For example, the E3 ubiquitin ligase NEDD4L promotes ferroptosis in ESCC by facilitating xCT ubiquitination, thereby inhibiting tumor cell growth and metastasis (Chen Z. et al., 2024). Ubiquitin-specific protease 2 (USP2) triggers ferroptosis in ESCC cells by removing Lys48-linked chains to stabilize nuclear receptor coactivator 4 (NCOA4) and prevent its degradation (Song J. et al., 2024). Sentrin-specific protease 1 (SENP1) inhibits ferroptosis and promotes the progression of HNSCC by regulating ACSL4 protein stability through small ubiquitin-like modifier (SUMO) modification (Xu et al., 2024). Increased expression of ubiquitin-conjugating enzyme 2T (UBE2T) has been found in tumor tissues of HNSCC patients and is associated with poor patient prognosis; knockdown of UBE2T inhibits HNSCC tumorigenesis and tumor growth. Further studies have shown that inhibition of UBE2T suppresses NF-κB signaling and induces ferroptosis in HNSCC (Cai et al., 2024). USP14 is among the most prominently upregulated deubiquitinating enzymes (DUBs) in HNSCC tissue samples and is associated with tumorigenesis and malignant progression of HNSCC. In addition to mediating the degradation of ferroptosis-related proteins through the autophagy-lysosomal degradation pathway, USP14 also inhibits FABP5 ubiquitination and degradation, thereby promoting ferroptosis in HNSCC cells. *In vivo* xenograft experiments confirmed that IU1, a small-molecule antagonist of USP14, can effectively attenuate cell growth, cisplatin resistance, invasion, and migration abilities in HNSCC (Qian et al., 2025). USP14 may therefore holds significant promise as a potential therapeutic target for HNSCC in future clinical applications.

In addition, several ferroptosis-related marker genes play important roles in the prognosis of HNSCC. For example, both TTC7B and PRMT5 are significantly overexpressed in HNSCC, and this increased expression is significantly associated with patients’ overall survival (OS), serving as an independent risk factor affecting OS. Based on functional enrichment analysis, TTC7B is associated with focal adhesion, cell migration, and immune infiltration (He R. et al., 2023). The DEGs modulated by PRMT5 exhibit significant enrichment in oncogenic signaling pathways, including IL-17 and p53. These genes further demonstrate strong associations with immune cell infiltration, m6A RNA methylation, FRG expression, and chemotherapeutic sensitivity (Zhang et al., 2024a). EMP1, belonging to the epithelial membrane protein family, demonstrates ferroptosis-promoting activity in HNSCC. Overexpression of EMP1 potentiates RSL3-mediated GPX4 suppression, thereby induces ferroptosis. EMP1 can also enhance the sensitivity of tumors to the targeted drug gefitinib (Wang et al., 2022); IL-6, secreted by tumor or immune cells, transcriptionally upregulates SLC7A11 expression by activating the JAK2/STAT3 pathway, inhibits lipid peroxidation, promotes tumor proliferation, EMT, and chemoresistance, and is associated with poor patient prognosis (Li M. et al., 2022). Its signaling is regulated by the negative feedback regulatory protein SOCS1, which inhibits IL-6 signal transduction by blocking STAT3 phosphorylation. In contrast, M2 exosome-derived Circ_0088494 enhances H3K4me1 modification of STEAP3 through KMT2D recruitment, thereby suppressing ferroptosis in CSCC (Yin et al., 2025). The relationship between cytokeratin 19 (CK19) and HNSCC prognosis remains inconclusive; however, CK19 expression levels are upregulated with the severity of oral mucosal epithelial dysplasia during OSCC development. Furthermore, CK19 knockdown modulates GPX4 and ACSL4 expression, promoting ferroptosis activation during OSCC progression (Rao et al., 2024). In OSCC, palmitoyl protein thioesterase 1 (PPT1) inhibits ferroptosis by modulating GPX expression, consequently promoting OSCC cell growth and proliferation (Luo et al., 2024). High cadherin CDH4 expression in OSCC specimens also effectively enhances OSCC cell proliferation and migration while reducing their sensitivity to ferroptosis, which correlates significantly with poor patient prognosis (Xie et al., 2023a). Guanosine triphosphate cyclohydrolase 1 (GCH1) drives ferroptosis by suppressing GPX4 and FSP1 expression in ESCC (Sakano et al., 2024).

Ferroptosis-related lncRNAs also serve as critical biomarkers for prognostic evaluation in HNSCC. Multiple ferroptosis-related lncRNAs (including AC010894.2, AC021087.4, HOTAIRM1, AC090246.1, ALMS1-IT1, AC099850.3, STARD4-AS1, LINC02158, AL512274.1, LINC01980, AATBC, and ELF3-AS1) significantly correlate with poor patient prognosis. Conversely, high expression of AC099850.4, AL512274.1, STARD4-AS1, and AL589986.2 is indicative of better clinical outcomes (Chu et al., 2019; Liang et al., 2020; Tang et al., 2020; Li H. et al., 2021; Li et al., 2024 J.; Lu R. et al., 2022).

### 3.2 Cuproptosis-related genes of HNSCC

Recent studies demonstrate that prognostic risk models based on cuproptosis-related genes (CRGs) exhibit robust predictive efficacy in HNSCC. Functionally, these CRGs are enriched in copper homeostasis and transport, mitochondrial oxidative stress, TCA cycle, p53 signaling, iron-sulfur cluster assembly, immune regulation, and DNA synthesis/repair, indicating their multifaceted roles in HNSCC progression through cuproptosis modulation.

Xu et al. constructed a risk model using 10 CRGs (NFE2L2, ATP7B, SLC31A1, FDX1, DLAT, PDHA1, MTF1, CDKN2A, DBT, and DLST) and validated its accuracy in predicting survival outcomes of HNSCC patients through independent datasets (Jiang X. et al., 2023). Another study identified 3 CRGs clusters based on 10 DEGs, then reclassified HNSCC patients into 3 gene clusters according to the expression of 8 DEGs (CDKN2A, PRELID2, ANP32B, MRPL47, CCDC59, WDR90, NLRX1, and KCNK6), and established a cuproptosis score (CS) system based on principal component analysis. These prognostic models consistently demonstrated strong associations with differential immune cell infiltration within the tumor microenvironment. Patient groups with favorable prognoses exhibited more significant immune cell infiltration (particularly CD4^+^ memory T cells) and better responses to immunotherapy. CDKN2A was identified as a CRG that is significantly upregulated in HNSCC tissues. As a key gene encoding the tumor suppressor protein p16INK4a, CDKN2A exhibits significantly high expression in HPV-positive patients, and its high expression level is significantly associated with longer overall survival (Padhi et al., 2017). Previous studies have established that the CDKN2A gene is frequently mutated in tumor suppressors and checkpoint mediators in HPV-negative HNSCC (Deneka et al., 2022). Additionally, CDKN2A is highly expressed in the epithelial cells of HNSCC and shows a significant positive correlation with the recruitment of immune cells, including CD8^+^ T cells, follicular helper T cells, and M1 macrophages (Padhi et al., 2017). Furthermore, silencing CDKN2A promotes autophagy and upregulates the autophagy markers LC3II and BECN1 (Yue et al., 2024). Therefore, CDKN2A plays an important role in the prognosis of HNSCC patients (Huang J. et al., 2022; Peng et al., 2023). Similarly, in the study of CRGs in ESCC, a total of 7 differentially expressed genes (FDX1, DLAT, LIAS, PDHB, MTF1, GL, and CDKN2A) were identified, among which DLAT and LIPT1 were respectively upregulated in cancers of different stages. Elevated expression levels of CDKN2A and PDHA1 were significantly correlated with enhanced overall survival, whereas reduced LIAS expression showed a positive association with favorable clinical outcomes.

Zhang et al. identified 14 CRGs that are significantly associated with the prognosis of HNSCC: Among these, ACLY, COX11, COX19, and PRKN exhibit negative correlations with OS, while ABCB1, BCL2, CDKN2A, CYP2D6, and DAPK2 show positive correlations with OS (Zhang et al., 2022b). COX11, COX19, and MT1E are involved in copper transport processes. The interaction between copper chelators and ABCB1 not only inhibits ABCB1-mediated transport but also suppresses ABCB1 expression (Ghosh et al., 2012). In mitochondrial homeostasis, PRKN and ACLY serve essential regulatory functions. Multiple genes, including CYP2D6, DAPK2, BCL2, RANTES, and IL-8, demonstrate connections to copper-dependent redox processes. Notably, these 14 genes exhibit significant enrichment in the olfactory transduction pathway. This finding is particularly relevant given the elevated copper concentrations observed in mammalian brain regions housing olfactory receptors (Horning and Trombley, 2001). Emerging evidence indicates that olfactory receptor-related genes contribute not only to sensory neurotransmission but also significantly influence pro-inflammatory responses and metastatic progression in cancer (Orecchioni et al., 2022). These observations suggest a potential mechanistic link between copper metabolism genes and olfactory receptor signaling in tumor biology. Zheng et al. selected 12-CRGs for OS prediction in HNSCC and inferred that among them, POLE, NTHL1, DNA2, ISCA2, and MTFR1L may have greater clinical application potential. ISCA2 is upregulated in HNSCC and is closely associated with the prognosis of HNSCC patients. ISCA2 serves as a critical mediator of Fe-S cluster biogenesis and contributes significantly to the development of multiple mitochondrial dysfunction syndromes (Weiler et al., 2020; Lebigot et al., 2021). Inhibition of ISCA2 can reduce HIF-α levels and also trigger ferroptosis through other signaling pathways (Green et al., 2022). In the human body, DNA2 is primarily localized within mitochondria, where it participates in the replication and repair of mitochondrial DNA. DNA2 can not only inhibit tumorigenesis by maintaining genomic integrity but also promote cancer cell survival by counteracting replication stress (Zheng et al., 2020). MTFR1L is a mitochondrial outer membrane-localized protein that regulates mitochondrial morphology and is essential for stress-induced AMPK-dependent mitochondrial fragmentation (Tilokani et al., 2022). Additionally, CD4^+^ T cell activation and antigen processing and presentation are suppressed in the high-risk group defined by the 12-CRGs signature (Zheng et al., 2023b).

Copper death-related lncRNAs also exert a significant influence on predicting the prognosis of HNSCC. Recent studies have identified multiple cuproptosis-associated lncRNAs that show good performance in the detection and prognostic prediction of HNSCC. For example, AL132800.1, LINC02901, lnc-FGF3-4, MYOSLID, FAM27E3, LINC02454, lncRNAMIR9-3HG, CDKN2A-DT, and SNHG16 are considered risk factors for HNSCC. In contrast, the upregulation of AC090587.1, AC012313, LINC01269, MAP4K3-DT, and THAP9-AS1 has been found to correlate with better prognosis in HNSCC patients (Xiong et al., 2019; Cheng et al., 2021; Li Y. J. et al., 2022; Li et al., 2022 X.; Zhao et al., 2022; Zhou L. et al., 2022; Han et al., 2023; Zheng et al., 2023c; Gong et al., 2024). Multiple studies have developed risk scoring systems based on cuproptosis-related lncRNAs, which exhibit strong predictive power for immune infiltration and high accuracy in survival prediction, thereby providing a theoretical foundation for risk stratification and personalized therapy in HNSCC patients. However, further mechanistic investigations remain limited, and future research should conduct in-depth analysis to validate the intricate associations between cuproptosis-related lncRNAs and HNSCC.

Emerging research indicates that integrating these dual predictive biomarkers significantly improves prognostic model performance and offers novel translational opportunities for developing precision oncology tools across multiple cancer types (Lv et al., 2020). Novel models based on ferroptosis/cuproptosis-related genes exhibit excellent potential for predicting the prognosis of multiple cancers (Li et al., 2023b; Zhang et al., 2023a). A prognostic model for HNSCC has been developed based on both ferroptosis/cuproptosis-related genes, which identified 12 key prognostic genes. Among these, NQO1, HSPA5, ATG5, G6PD, AURKA, CDKN2A, MAPK9, GABARAPL2, and Cav1 have also been frequently referenced in other studies. Inhibition of AURKA markedly attenuates the proliferative and migratory potential of Cal27 and CNE2 cell lines. In addition to its established role in tumor cell ferroptosis induction, AURKA may also be associated with CRGs such as DBT, DLST, and LIAS; the underlying mechanisms of their interaction require further investigation (Jia et al., 2024). In addition to the genes already mentioned above, NQO1, HSPA5, and ATG5 are all overexpressed in HNSCC and associated with poor prognosis in HNSCC patients, making them promising targets for HNSCC treatment (Hamada et al., 2021; Lundberg et al., 2021; Shi et al., 2022). GABARAPL2, MAPK9, and CDKN2A are all related to autophagy, further illustrating the complex connections among autophagy, ferroptosis, and cuproptosis in HNSCC (Ren et al., 2021). In a recent study, Liu et al. integrated a large OSCC single-cell transcriptome dataset and used ferroptosis suppressor genes and cuproptosis suppressor genes as markers to identify the Epi_2 subtype associated with the metal-dependent cell death resistance (MCDR) score in malignant cells within metastatic primary tumors (mPT). Immunohistochemical analysis revealed that the Epi_2 signature markers CTSV and GPX4, along with cells co-expressing CTSV, GPX4, and CDKN2A, were significantly more highly expressed in mPT than in nPT. Moreover, OSCC patients with high Epi_2 characteristics may exhibit resistance to immunotherapy and anti-EGFR therapy (Liu X.-H. et al., 2025).

As comprehensively documented in the Table 1, the prognostic model constructed by integrating ferroptosis/cuproptosis-related genes provides a significant breakthrough for the precise prediction and optimization of treatment strategies in HNSCC. Through systematic identification of pivotal hub genes (NQO1, AURKA, and CDKN2A), this study has not only elucidated the synergistic mechanisms underlying the ferroptosis-cuproptosis regulatory network in HNSCC progression, but also characterized the clinically resistant Epi_2 molecular subtype with distinct therapeutic vulnerabilities. These findings establish a theoretical foundation for developing targeted therapies based on the regulation of metal-dependent cell death. Future research should further investigate the interaction mechanisms among key genes and advance the clinical application of this model in predicting resistance to immunotherapy and targeted therapy.

**TABLE 1:** Prognostic impact of genes associated with ferroptosis/cuproptosis in HNSCC.

Type of cell death	Genes involved	Outcome	Functional roles	References
Ferroptosis	SLC7A11, AURKA, TRIB3, AKR1C3, CAV1	Overall survival (OS) prediction	AURKA: Upregulated in head and neck squamous cell carcinoma (HNSCC) to inhibit ferroptosis, which is associated with poor prognosis and cisplatin resistance TRIB3: Mainly promotes HNSCC progression by inhibiting ferroptosis AKR1C3: Reduces lipid peroxide levels CAV1: Decreases the expression of NOX1 and ACSL4, while increasing the expression of FTH1 and GPX4; this reduces HNSCC sensitivity to ferroptosis and stabilizes the ATP7A protein	Li et al. (2021a)
Ferroptosis	ATG5, AURKA, CAV1, FTH1, SLC3A2, SLC7A11, SOCS1, etc.	1. OS prediction 2. Construction of cluster subgroups based on the tumor microenvironment (TME)	ATG5: An autophagy-related gene that drives ferroptosis in HNSCC and inhibits CD8^+^ T cells G6PD: Promotes disease progression in oral squamous cell carcinoma (OSCC) patients SOCS1: Downregulates SLC7A11 expression and glutathione (GSH) levels, thereby increasing ESCC sensitivity to ferroptosis	Huang et al. (2022b)
Ferroptosis	GOT1, AURKA, EGFR, CAV1, CA9, GRIA3, TRIB3, AKR1C3, TTPA, PPARG	OS prediction	CA9: Upregulated in OSCC to inhibit ferroptosis EGFR: Upregulated in HNSCC; decreases nasopharyngeal carcinoma (NPC) sensitivity to ferroptosis and regulates autophagy	Tang et al. (2024b)
Ferroptosis	NEDD4L	Induces ferroptosis in ESCC	Promotes XCT ubiquitination to induce ferroptosis	Chen et al. (2024d)
Ferroptosis	USP2	Induces ferroptosis in ESCC	Removes Lys48-linked polyubiquitin chains to stabilize NCOA4 and prevent its degradation, thereby triggering ferroptosis	Song et al. (2024b)
Ferroptosis	SENP1	Inhibits ferroptosis in HNSCC	Regulates ACSL4 protein stability via small ubiquitin-like modifier (SUMO) modification to suppress ferroptosis	Xu et al. (2024)
Ferroptosis	UBE2T	Inhibits ferroptosis in HNSCC	Activates the NF-κB pathway to inhibit ferroptosis	Cai et al. (2024)
Ferroptosis	USP14	Induces ferroptosis in HNSCC	Regulates the degradation of ferroptosis-related proteins; inhibits FABP5 ubiquitination and degradation to induce ferroptosis	Qian et al. (2025)
Ferroptosis	TTC7B, PRMT5	Overexpressed in HNSCC	Serve as independent risk factors affecting HNSCC patients’ OS.	He et al. (2023b)
Ferroptosis	EMP1	Induces ferroptosis in HNSCC	Potentiates RSL3-mediated GPX4 suppression; enhances tumor sensitivity to the targeted drug gefitinib	Wang et al. (2022)
Ferroptosis	CK19	Induces ferroptosis in OSCC	Modulates GPX4 and ACSL4 expression to trigger ferroptosis	Rao et al. (2024)
Ferroptosis	PPT1	Inhibits ferroptosis in OSCC	Modulates glutathione peroxidase (GPX) expression to suppress ferroptosis	Luo et al. (2024)
Ferroptosis	GCH1	Induces ferroptosis in ESCC	Suppresses GPX4 and FSP1 expression to induce ferroptosis	Sakano et al. (2024)
Cuproptosis	NFE2L2, ATP7B, SLC31A1, FDX1, DLAT, PDHA1, MTF1, CDKN2A, DBT, DLST, PRELID2, ANP32B, MRPL47, etc.	1. OS prediction 2. Exhibits strong associations with differential immune cell infiltration in the TME	CDKN2A: Encodes the tumor suppressor protein p16INK4a; highly expressed in human papillomavirus (HPV)-positive patients, with its expression level significantly correlated with OS; recruits immune cells and inhibits tumor cell autophagy	Jiang et al., 2023b; Huang et al., 2022a
Cuproptosis	FDX1, DLAT, LIAS, PDHB, MTF1, GL, CDKN2A	1. OS prediction 2. Identification of two ESCC molecular subtypes based on the expression profiles of 10 cuproptosis-related genes (CUGs)	PDHA1: Upregulated in ESCC and associated with better OS. DLAT: Upregulated in stage III ESCC. LIPT1: Upregulated in ESCC with N0/N1 lymph node status LIAS: Low expression is associated with better clinical outcomes in ESCC patients Milciclib may inhibit the proliferation and migration of KYSE150 and KYSE510 ESCC cells by targeting CDKN2A	Wu et al. (2023)
Cuproptosis	ACLY, COX11, COX19, PRKN, ABCB1, BCL2, CDKN2A, CYP2D6, DAPK2, etc.	OS prediction	COX11, COX19, and MT1E are involved in copper transport; the interaction between copper chelators and ABCB1 not only inhibits ABCB1-mediated transport but also suppresses ABCB1 expression PRKN and ACLY are associated with the regulation of mitochondrial homeostasis	Zhang et al. (2022b)
Cuproptosis	POLE, NTHL1, DNA2, ISCA2, MTFR1L	OS prediction	ISCA2: Assembles iron-sulfur (Fe-S) clusters, affects mitochondrial function, and reduces hypoxia-inducible factor-α (HIF-α) levels DNA2 and MTFR1L are associated with the regulation of mitochondrial homeostasis	Zheng et al. (2023b)
Ferroptosis/Cuproptosis	NQO1, HSPA5, ATG5, G6PD, AURKA, CDKN2A, MAPK9, GABARAPL2, CAV1, etc.	OS prediction	AURKA: Upregulated in HNSCC to inhibit ferroptosis; associated with CUGs such as DBT, DLST, and LIAS. NQO1, HSPA5, and ATG5 are overexpressed in HNSCC and associated with poor prognosis GABARAPL2, MAPK9, and CDKN2A are involved in autophagy	Jia et al. (2024)
Ferroptosis/Cuproptosis	CTSV, GPX4 CTSV, GPX4, CDKN2A	Predicting immunotherapy resistance and anti-EGFR therapy resistance in OSCC patients with high Epi_2 signature	Epi_2 signature markers (CTSV, GPX4) and cells co-expressing CTSV, GPX4, and CDKN2A are significantly more highly expressed in matched primary tumors (mPT) than in non-primary tumors (nPT)	Liu et al. (2025b)
Ferroptosis/Autophagy	DDIT4	Overexpresses and induces ferroptosis in HNSCC	Promotes the expression of HIF-1α, VEGF, and vimentin Impact tumor immunity Renders cancer cells sensitive to autophagy by inhibiting the mTOR signaling pathway in HT22 cells	Zhang et al. (2023c); Zhang et al. (2023b)
Ferroptosis/Autophagy	CISD2	1. OS prediction 2. Overexpression linked to tumor drug resistance	Inhibits the function of NAF-1, inducing cellular autophagy, possibly via activating ferritinophagy to increase intracellular labile iron levels	Li et al. (2024e); Kim et al. (2018)

## 4 Potential interplay and clinical association between F/CRGs in HNSCC therapy

### 4.1 Chemotherapy and targeted drugs

Studies have shown that ferroptosis plays an important role in enhancing the efficacy of chemotherapy for HNSCC and overcoming drug resistance, particularly with alkylating chemotherapeutic agents. Multiple studies have confirmed that combining alkylating agents such as cisplatin with the ferroptosis inducer erastin can significantly improve anticancer efficacy against HNSCC. The primary mechanism involves inhibiting SLC7A11, thereby increasing the sensitivity of cisplatin-resistant HNSCC cells to ferroptosis (Roh et al., 2016). Similarly, in PDAC, co-administration of dihydroartemisinin (DHA) and cisplatin enhances cytotoxicity through the ferroptosis pathway (Du J. et al., 2021). Additionally, temozolomide (TMZ) combined with RSL3 significantly inhibits glioblastoma cell proliferation by inducing ferroptosis (Song et al., 2021). Research has also demonstrated that silencing the AEBP1 gene resensitizes cisplatin-resistant OCSS cells to ferroptosis by activating the JNK/p38/ERK pathway (Zhou Q. et al., 2022), while targeting the NRF2/ABCC1 axis effectively reverses chemoresistance in gliomas (De Souza et al., 2022). Regarding cuproptosis mechanisms, research has revealed that cisplatin utilizes the copper transporter CTR1 for cellular uptake, with tumor CTR1 expression levels serving as a predictive biomarker for platinum-based chemotherapy response (Ishida et al., 2002; Kim et al., 2014). Meanwhile, recent studies have not only revealed that extracellular vesicles (EVs) in HNSCC mediate cisplatin resistance by upregulating ATP7B, but also demonstrated that the copper chelator ammonium tetrathio molybdate (TM) can enhance cisplatin efficacy in HNSCC by reducing ATP7B expression (Ogawa et al., 2024). These findings suggest that targeting the ATP7B pathway may overcome drug resistance through cuproptosis modulation. Additionally, the copper ionophore elesclomol combined with copper ions (CuCl_2_) triggers cuproptosis in prostate cancer cells and enhances docetaxel sensitivity by upregulating DLAT protein and inhibiting the mTOR pathway (Wen et al., 2023). The copper complex CuET can also reverse cisplatin resistance in A549/DDP cells by downregulating FDX1 expression (Lu Y. et al., 2022).

Ferroptosis inducers (FINs) are currently classified based on various mechanisms, including those targeting the Xc^−^ system, GPX4 inhibitors or degraders, compounds that deplete coenzyme Q10, and lipid peroxidation inducers (for details, refer to the review by JIN X) (Jin et al., 2024). System Xc^−^-inhibitors effectively induce ferroptosis in tumor cells by blocking cystine uptake, depleting GSH, and promoting lipid peroxidation. Representative drugs such as erastin and its derivatives, sorafenib, and natural compounds (TalaA, 18β-glycyrrhetinic acid) have demonstrated antitumor activity in various cancers (e.g., HCC, DLBCL, CRC) by inhibiting the SLC7A11 or VDAC pathway (Li Z. et al., 2021; Li et al., 2022 H.; Wen et al., 2021). Blocking SLC7A11 with Erastin significantly reduces the malignant phenotype of ESCC cells and downregulates key ferroptosis-related molecules GPX4 and DHODH (Li W.-T. et al., 2024); GPX4 inhibitors, by directly blocking the lipid repair pathway, may induce ferroptosis more efficiently than system Xc^−^ inhibitors. Synthetic compounds such as RSL3 and ML162/210, along with natural derivatives (e.g., Oridonin A), induce the accumulation of lipid peroxidation by inhibiting or degrading GPX4. Novel GPX4 degraders (e.g., DC-2, 8e) have further improved targeting and safety, demonstrating potent antitumor activity in various cancer models (Randolph et al., 2023; Wang C. et al., 2023; Wang et al., 2023 H). The chemotherapeutic drug cisplatin can also inhibit GPX4, thereby inducing ferroptosis in A549 and HCT116 cells to suppress their proliferation (Shi Y. et al., 2019; Huang J.-Y. et al., 2024). LncRNA (TMEM44-AS1) is positively correlated with GPX4 expression; it can bind to the RNA-binding protein IGF2BP2 to enhance the stability of GPX4 mRNA, thereby affecting ferroptosis and regulating the malignant progression of ESCC (Yang R. et al., 2023). While class III FINs targeting the coenzyme Q10-FSP1 pathway enhance tumor therapy by blocking the antioxidant defense system. For example, iFSP1/icFSP1 directly inhibits FSP1-mediated coenzyme Q10 regeneration and synergizes with GPX4 inhibitors to significantly induce ferroptosis. FSP1 is significantly upregulated in recurrent tissues of cisplatin-resistant HNSCC, accompanied by activation of lipid metabolism genes. Inhibition of FSP1 blocks the FSP1/ACSL4 axis, significantly suppressing cancer stem cell (CSC) activity and metastatic capacity (Wu Y.-C. et al., 2024). Natural compounds (e.g., curcumin, andrographolide) can dual inhibit GPX4 and FSP1, exhibiting anti-tumor potential in colorectal cancer (CRC) (Miyazaki et al., 2023). Statins are also typical class III FINs, which inhibit the mevalonate (MVA) pathway and downregulate GPX4. The use of statins may protect HNSCC patients against adverse outcomes, particularly HPV-positive patients (Getz et al., 2021), and may enhance responses to PD-1 checkpoint blockade and other HNSCC immunotherapies by modifying the HNSCC tumor immune microenvironment (Kansamurine modelsl et al., 2023). The relationship between these drugs and ferroptosis in HNSCC requires further investigation; Class IV FINs targeting lipid peroxidation enhance antitumor effects through iron overload and oxidative stress. For instance, artemisinin derivatives (e.g., dihydroartemisinin) induce ferritin autophagy, leading to the release of labile iron (Chen et al., 2020). Artesunate, an artemisinin derivative, increases ROS, reduces GSH, triggers ferroptosis, and inhibits the proliferation of HNSCC cells (Roh et al., 2017). The transcription factor NRF2 functions as a key negative regulator of ferroptosis. As demonstrated by compounds including Erianin, cetuximab, juglone, and shikonin, which induce ferroptosis by inhibiting the NRF2/HO-1 pathway or bidirectionally modulating HO-1, leading to elevated ROS, Fe^2+^, and lipid peroxidation in various cancer models (Xiang et al., 2021; Yang et al., 2021; Ni et al., 2023). Notably, the NRF2 inhibitor Brusatol provides a compelling mechanistic example: it triggers ferroptosis in HNSCC models (including ESCC and OSCC) by promoting NRF2 degradation, which subsequently represses the expression of key genes like GCLC, thereby depleting GSH, enlarging the labile iron pool, and driving the accumulation of lethal lipid ROS (Zhu et al., 2023; Qi et al., 2025). The efficacy of these NRF2-targeting agents underscores the translational potential of inducing ferroptosis for HNSCC treatment. Baicalin is a flavonoid compound extracted and isolated from the dried roots of Scutellaria baicalensis. It directly inhibits the expression of FTH1 in OSCC cells and effectively promotes ferroptosis by targeting FTH1, while also inhibiting proliferation and EMT (Wen et al., 2024).

Although relatively few studies have focused on discovering drugs that induce cuproptosis for cancer treatment, it is anticipated that as the underlying mechanisms of cuproptosis are gradually illuminate, more cuproptosis-targeting compounds will be developed to advance cancer therapy in the future (Jin et al., 2024). In recent years, research has revealed that 4-OctylItaconate (an antioxidant that activates NRF2) can target GAPDH to inhibit glycolysis and promote cuproptosis in colorectal cancer cells (Yang W. et al., 2023). DSF/Cu has also demonstrated significant clinical potential in the treatment of NPC: it can induce ferroptosis in tumor cells through the ROS/MAPK-p53 pathway and promote apoptosis and necrosis of α-SMA-positive cancer-associated fibroblasts (CAFs). Additionally, the combination of DSF/Cu with cisplatin synergistically inhibits tumor growth with good *in vivo* tolerance. These findings provide a novel adjuvant strategy for NPC treatment and demonstrate significant translational application value (Li et al., 2020). A study further revealed the synergistic mechanism between cuproptosis and ferroptosis: sorafenib and erastin promote both copper-dependent protein lipoylation and ferroptosis in HCC cells by inhibiting FDX1 degradation and GSH synthesis (Wang W. et al., 2023). This offers new insights into combination strategies involving targeted drugs (such as anti-EGFR or BCL-2 inhibitors) with cuproptosis inducers, which are particularly applicable to drug-resistant tumors with hyperactive mitochondrial metabolism, though further clinical validation is required.

These important findings indicate that targeting ferroptosis and cuproptosis-related pathways may serve as an effective strategy to overcome HNSCC chemotherapy resistance and develop novel targeted therapies. Future research should focus on exploring common regulatory targets of the two cell death modalities (such as GSH metabolism and oxidative stress pathways) and further illuminate the mechanisms underlying their synergistic interactions. The coordinated modulation of ferroptosis and cuproptosis pathways represents a promising therapeutic strategy that may substantially improve the anticancer efficacy of existing drugs, more effectively reverse tumor drug resistance, and provide a solid theoretical foundation for developing innovative combination therapeutic regimens. This will become a crucial research direction for overcoming the therapeutic bottlenecks in HNSCC.

### 4.2 Radiotherapy

Ionizing radiation effectively triggers ferroptotic cell death, which is also a key part of the anticancer effects brought about by radiation therapy (RT). RT induces ferroptosis through the generation of ROS, a process primarily mediated by lipid peroxidation reactions. The underlying mechanism involves ROS reacting with PUFAs via electron transfer to form lipid peroxyl radicals (LOO·) and their derivatives, lipid hydroperoxides (LOOH). RT also upregulates ACSL4 to promote the production of PUFAs-containing phospholipids, reduces GSH levels, impairs GPX4, and thereby promotes ferroptosis (Ye et al., 2020). Multiple studies have demonstrated that inhibiting key ferroptosis regulatory factors can significantly reduce RT sensitivity. For instance, ferroptosis inducers synergize with RT by depleting GSH or disrupting lysosomal iron sequestration (Pan et al., 2019; Ma et al., 2021). Concurrently, autophagy activation and ferroptosis engage in cross-regulation to further enhance RT sensitivity, and ferroptosis also represents a novel intersection between immunotherapy and RT (Zheng et al., 2023a). RT plays a critical anti-cancer role by inducing ferroptosis, but cancer cells can develop resistance through the upregulation of GPX4 and FSP1 (Lang et al., 2019; Lei et al., 2020). Additionally, statins (such as atorvastatin) inactivate GPX4 by inhibiting the MVA pathway, reversing RT resistance and restoring ferroptosis sensitivity (Wang L. et al., 2025).

In recent years, significant breakthroughs have been made in research into the role of ferroptosis regulatory mechanisms in HNSCC radiotherapy, with multiple key regulatory targets and their molecular mechanisms gradually being illuminate. Studies have demonstrated that the histone demethylase JMJD2A promotes GPX4 expression by regulating Smarca4, thereby suppressing RT-induced ferroptosis. This molecular pathway critically regulates both tumor aggressiveness and immune escape mechanisms in ESCC (Su et al., 2024). Glutathione S-transferase mu3 (GSTM3) interacts with GPX4 to inhibit its expression. Combining RT with GSTM3 modulation synergistically enhances NPC radiosensitivity and inhibits tumor growth (Chen Y. et al., 2024). Polo-like kinase 1 (PLK1) influences NADPH and GSH levels by regulating the pentose phosphate pathway (PPP); its downregulation promotes ferroptosis and increases sensitivity to radiotherapy and chemotherapy (Zhao M. et al., 2023). Similarly, the mitochondria-targeted tamoxifen analog MitoTam can effectively induce ferroptosis in HNSCC cells by inhibiting the antioxidant defense system of RT-resistant cells, thereby significantly enhancing the sensitivity of tumor cells to RT treatment (Reinema et al., 2024). Furthermore, the E3 ubiquitin ligase NEDD4L promotes KLF5 ubiquitination and subsequent proteasomal degradation, relieving its inhibitory effect on ferroptosis and consequently enhancing the radiosensitivity of ESCC cells (Chen J. et al., 2024). In terms of epigenetic regulation, the m6A demethylase FTO inhibits radiation-induced ferroptosis through modification of OTUB1 transcripts and is significantly upregulated in radioresistant NPC (Huang et al., 2023). The STC2-PRMT5 signaling axis not only suppresses SLC7A11-mediated ferroptosis in a PRMT5-dependent manner but also confers tumor cell radioresistance by enhancing DNA damage repair pathways such as homologous recombination and non-homologous end joining. High expression of this axis in radioresistant ESCC cells is significantly associated with poor patient prognosis (Jiang K. et al., 2023). These findings not only reveal the central role of ferroptosis in regulating the radiosensitivity of HNSCC, but also provide multiple potential intervention targets for developing novel radiosensitization strategies. Additionally, multiple studies have demonstrated that ferroptosis-related miRNAs play a crucial role in modulating the radiosensitivity of NPC cells. For instance, MicroRNA-372 enhances NPC radiosensitivity by activating the PBK-dependent p53 signaling pathway while inhibiting cell invasion and metastasis (Wang et al., 2019). Both miR-124 and miR-9 may promote NPC radiosensitivity by targeting programmed cell death protein 6 (PDCD6) and suppressing the expression of junctional adhesion molecule A (JAMA) (Chen et al., 2019; Tian et al., 2020). In contrast, miR-182-5p promotes NPC radioresistance through regulating BNIP3 expression (He et al., 2020). In the context of combined immunotherapy, resistance is induced in HNSCC when RT upregulates the SLC1A5 transporter to increase glutamine uptake. Dual blockade of glutamine and CD47 not only synergistically enhances RT-induced ferroptosis but also significantly improves the tumor microenvironment, thereby offering a novel strategy for the radio-metabolic-immune combined therapy of HNSCC (Song A. et al., 2024).

The relationship between RT and cuproptosis is also highly significant. RT induces cuproptosis in cancer cells, with its molecular hallmarks being the marked depletion of lipoylated proteins and Fe-S cluster proteins in patients’ tumor tissues; the loss of both proteins serves as a typical pathological signature of cuproptosis. The mechanistic basis involves RT-induced mitochondrial copper accumulation through coordinated upregulation of CTR1 transporter and depletion of mitochondrial GSH, ultimately initiating cuproptosis. Bulk RNA sequencing of radioresistant ESCC cell lines and single-cell RNA-seq analysis of primary esophageal tumor specimens, revealed a significant correlation between radioresistance and decreased expression of the oxidative stress regulator BACH1. Downregulation of BACH1 relieves its transcriptional repression of copper-chelating metallothioneins (MT1E/MT1X), consequently reducing intracellular cuproptosis levels and ultimately contributing to the development of resistance (Lei et al., 2025). Moreover, glutaminase 2 (GLS2) inhibition synergizes with copper to reprogram the TCA cycle, which can be utilized for radiosensitization under copper poisoning conditions in ESCC (Jing et al., 2025). Recent studies have demonstrated that copper metabolism MURR1 domain 10 (COMMD10), a protein involved in copper metabolism, impedes the HIF1α/CP loop, thereby enhancing ferroptosis and radiosensitivity by disrupting Cu-Fe homeostasis in HCC (Yang et al., 2022). Additionally, various copper-based nanocomposites have recently been developed to increase the sensitivity of RT. For instance, Shao et al. engineered an innovative theranostic nanoplatform that synergizes with low-dose radiotherapy (LDRT, i.e., 0.5–2 Gy) to induce cuproptosis for the treatment of HCC (Shao et al., 2024). CuGI@CM can selectively eliminate hypoxic tumors by boosting ROS production and facilitating oligomerization of lipoylated proteins in the tricarboxylic acid cycle, and when combined with RT, it significantly enhances the therapeutic efficacy (Liu J. et al., 2025). These clues suggest that targeting Cu or Cu-related proteins may represent a novel radiosensitization strategy. Furthermore, serum Cu levels in cancer patients are negatively correlated with RT response, indicating that serum Cu also serves as an effective indicator for monitoring RT efficacy (Tessmer et al., 1973). Concurrently, due to the unique emission properties of copper isotopes, copper-based radiopharmaceuticals have achieved numerous recent successful applications not only in PET and SPECT imaging but also in RT and radioimmunotherapy (Krasnovskaya et al., 2023). With future breakthroughs in isotope production and integration with nanomaterials, the effectiveness and enormous potential of copper-containing radiopharmaceuticals as both imaging and therapeutic agents will undoubtedly be further enhanced.

In conclusion, ferroptosis and cuproptosis exhibit significant potential for synergistic enhancement in HNSCC radiotherapy. Radiotherapy triggers these two types of programmed cell death by inducing ROS production, lipid peroxidation, and mitochondrial copper overload; however, tumor cells can also develop resistance through multiple compensatory mechanisms. Combined targeting of key regulators of ferroptosis (e.g., GPX4, SLC7A11) and cuproptosis-related pathways (e.g., CTR1, BACH1) significantly enhances radiosensitivity. The development of novel nanomaterials provides an innovative approach for the simultaneous induction of ferroptosis and cuproptosis, and their synergistic effect with RT has been validated in various tumor models. For future HNSCC combination chemotherapy, in addition to developing small-molecule inhibitors or nanoformulations that dual-target ferroptosis and cuproptosis, integration with immunotherapy is expected to enhance tumor immunogenicity. Concurrently, serum copper/iron metabolism markers should be explored to enable precise treatment monitoring. As our understanding of the interaction mechanisms between ferroptosis and cuproptosis deepens, combined therapeutic regimens targeting these two forms of cell death will emerge as a crucial development direction for the individualized treatment of HNSCC.

### 4.3 Immunotherapy

Ferroptosis in cancer cells dynamically interacts with immune components in the tumor microenvironment (TME). This process modulates immune responses by altering cytokines and immune cell recruitment. Activated lymphocytes and macrophages can also promote ferroptosis via ROS and cytokine signaling. For example, tumor-associated neutrophils (TANs) transfer peroxidase-containing granules to tumor cells, resulting in the accumulation of iron-dependent lipid peroxidation products and subsequent induction of ferroptosis (Yee et al., 2020). Thus creating a tumor-suppressive loop.

Ferroptosis induction profoundly alters the tumor immune landscape by depleting immunosuppressive populations - particularly myeloid-derived suppressor cells (MDSCs) and M2-polarized tumor-associated macrophages (M2TAMs), while enhancing the recruitment and activation of CD4^+^ and CD8+T cells (Zhao Y. et al., 2023). Multiple ferroptosis modulators impact tumor microenvironment composition. Research by Efimova et al. demonstrates that RSL3 treatment potentiates dendritic cell proliferation, activation, and immune function in murine models of fibrosarcoma and glioma through time-dependent mechanisms. This immunostimulatory effect appears mediated by tumor-derived ATP and HMGB1 release (Efimova et al., 2020). GPX4 not only safeguards activated regulatory T cells (Tregs) against ferroptosis but also contributes to the survival and proliferation of CD4^+^ and CD8^+^ T cells. Through these dual roles, GPX4 plays a pivotal part in suppressing anti-tumor immune responses (Xu et al., 2021; Yao et al., 2021). N6F11-induced ferroptosis promotes HMGB1 release, activates CD8^+^ T cell-mediated anti-tumor immunity, and synergizes with PD-L1 inhibitors to enhance therapeutic efficacy against KRAS/TP53 mutant pancreatic cancer, thus providing a new strategy for tumor immunotherapy (Li et al., 2023a). Beyond inducing ferroptosis by suppressing system Xc^−^ and decreasing GSH synthesis, sorafenib can also stimulate tumor-associated macrophages (TAMs) to secrete IL-12, which in turn promotes the apoptosis of cancer cells. When combined with the generation of mCAR T cells, this exerts an anti-tumor effect in mouse models of HCC (Wu et al., 2019). The upregulation of SLC2A3 in OSCC cells is associated with increased immune infiltration and poor clinical outcomes. It may promote tumor development in the tumor microenvironment by triggering ferroptosis, which negatively regulates the proliferation and function of CD8^+^ T cells (Jiang W. et al., 2024; Wu J. E. et al., 2024).

PD-1 and PD-L1 have garnered significant attention for their roles within the tumor microenvironment in recent years. Recent studies demonstrate that PD-L1 immunotherapy-activated CD8^+^ T lymphocytes release IFN-γ, which suppresses expression of the Xc^−^ system components SLC3A2 and SLC7A11. When combined with tumor microenvironment-derived arachidonic acid, this T cell-mediated response upregulates ACSL4 activity, ultimately triggering immunogenic ferroptosis in tumor cells (Liao et al., 2022). Multiple research investigations focusing on the combination of targeted ferroptosis and immune checkpoint blockade (ICB) therapy have indicated that triggering ferroptosis within tumor cells alongside treatment with anti-PD-1 antibodies yields a robust anti-tumor efficacy (Yang F. et al., 2023). Those HNSCC patients with high PD-L1 expression show reduced ROS levels and suppressed ferroptosis. Mechanistic investigations have revealed that PD-L1 markedly strengthens the resistance of HNSCC cells to ferroptosis through the activation of the SOD2-mediated antioxidant pathway. This finding implies that targeted modulation of the inherent regulatory role of PD-L1 could serve as a promising approach to enhance therapeutic effectiveness (Feng et al., 2024). Studies by Chung et al. have revealed that ferroptosis regulates PD-L1 expression in HNSCC through a dual mechanism: on the one hand, PD-L1 is upregulated via the ROS-driven NF-κB signaling pathway (a membrane damage-independent pathway), and on the other hand, immune checkpoint molecule expression is promoted through ferroptosis stress-induced calcium influx (a membrane damage-dependent pathway). Notably, this ferroptosis-PD-L1 regulatory axis is more pronounced in HPV-negative HNSCC, suggesting that ferroptosis characteristics may serve as a predictive marker for immune therapy response in HPV-negative patients (Chung et al., 2023). Recent studies have found that thioredoxin reductase (TXNRD1) can regulate PD-L1 transcription by binding to ribonucleotide reductase (RRM2) and is involved in maintaining redox balance to inhibit the ferroptosis process. HNSCC with high TXNRD1 expression exhibit characteristics of NRF2 activation, PD-L1 upregulation, and resistance to PD-1 inhibitors. The TXNRD1 inhibitor Auranofin promotes tumor cell death while downregulating PD-L1 expression and increasing CD8^+^ T cell infiltration, demonstrating a potent synergistic anti-tumor effect (Hsieh et al., 2024). Researchers like Yong developed a novel compound, B2, with enhanced mitochondrial targeting ability and antitumor activity by integrating the mitochondrial targeting group triphenylphosphine (TPP) with the DHODH inhibitor BRQ. This compound specifically inhibits the expression of DHODH, reduces the mitochondrial cristae structure, and effectively triggers ferroptosis in multiple cancer cell lines. Further research revealed that B2 can also regulate the expression levels of MTHFD2 and LACTB, resulting in a significant downregulation of PD - L1 and alleviating the tumor immunosuppressive microenvironment (Hai et al., 2024). These findings not only illuminate the molecular link between ferroptosis and tumor immune evasion but also suggest a potential therapeutic strategy of combining ferroptosis inducers with immune checkpoint inhibitors (ICIs) for HPV-negative HNSCC.

Currently, research into the interaction mechanisms between cuproptosis and HNSCC immunotherapy remains in the preliminary exploration stage, yet this field demonstrates significant translational medical potential. Current evidence suggests that tumor cells undergoing cuproptosis release a variety of damage-associated molecular patterns (DAMPs), including ATP, HMGB1, and CRT, which facilitate the maturation of DCs and the activation of CD8^+^ T cells (Guo et al., 2025). Cuproptosis also activates the tumor antigen presentation process by regulating the cGAS-STING signaling pathway, subsequently facilitating the release of inflammatory mediators (Jiang et al., 2022). Notably, copper increases PD-L1 expression at both transcriptional and protein levels in malignant tumors such as neuroblastoma cells, thereby influencing tumor immune escape mechanisms (Zhang et al., 2024b). Copper chelators (e.g., DC or TEPA) inhibits STAT3 and EGFR phosphorylation, subsequently promoting ubiquitin-dependent PD-L1 proteasomal degradation. Additionally, copper chelation of lysyl oxidase-like 4 (LOXL4) can effectively eliminate IFN-induced PD-L1 expression (Tan et al., 2021). Furthermore, copper chelators are capable of boosting the infiltration of CD8^+^ T cells and NK cells, resulting in significant suppression of tumor growth (Guo et al., 2025). The DSF/Cu combination has been shown to exhibit potent anti-tumor effects. In the context of immunotherapy, DSF/Cu treatment promotes the activation and maturation of DCs, particularly when combined with CD47 blockers. This not only increases the cytotoxic activity of CD8^+^ T cells but also upregulates macrophage function in HCC (Gao et al., 2022). Recent studies have extensively investigated ferroptosis/cuproptosis-related compounds or components, which exhibit notable anti-cancer effects in HNSCC, as summarized in Table 2.

**TABLE 2:** Anti-cancer effects of compounds related to ferroptosis/cuproptosis in HNSCC.

Compound	Cell lines	Therapeutic effect in HNSCC	Mechanism of action	References
Ferroptosis				
Erastin	HN3, HN4, HN9; KYSE30, KYSE150, KYSE180, KYSE510, TE1, TE10, Het-1A	Overcoming cisplatin resistance in HNSCC cells Suppresses the malignant phenotype of ESCC.	Inhibiting system Xc^−^ blocks cystine uptake and depletes glutathione, thereby promoting tumor cell ferroptosis	Roh et al., 2016; Li et al., 2024d
RSL3	CAL27, CAL33, SCC9; CNE-2; MCA205,GL261	Induces ferroptosis in HNSCC cells Sensitizes EGFR inhibition-resistant HNSCC to cetuximab Potentiates dendritic cell proliferation, activation, and immune function in murine models of fibrosarcoma and glioma	Directly inhibits GPX4 activity, preventing lipid peroxide clearance Enhances cetuximab’s antitumor effect by blocking the EREG/GPX4 axis Promotes the release of tumor-derived ATP and HMGB1	Jehl et al., 2023; Liu et al., 2022b; Efimova et al., 2020
Baicalin	Cal27, SCC25	Induces ferroptosis and to inhibits OSCC proliferation and EMT.	Suppresses FTH1 activity to increase labile Fe^2+^	Wen et al. (2024)
iFSP1	HSC3, HSC4	Triggers ferroptosis in HNSCC cells	Suppresses the FSP1/ACSL4 axis, blocks FSP1-mediated CoQ10 regeneration, and synergizes with GPX4 inhibitors to induce ferroptosis	Wu et al. (2024b)
Brusatol	KYSE150,KYSE450; Cal-27	Triggers ferroptosis in OSCC and ESCC cells	Inhibits downstream pathways (e.g., NRF2/HO-1, Nrf2/GCLC), leading to intracellular GSH depletion, accumulation of Fe^2+^ and ROS, and induction of ferroptosis	Zhu et al., 2023; Qi et al., 2025
Statins (atorvastatin, simvastatin)	MOC1, TC-1	Improves survival of HPV^+^ HNSCC patients Enhances response to anti-PD-1 immunotherapy	Inhibits mevalonate (MVA) pathway, reducing GPX4 expression and sensitizing cells to ferroptosis Modulates tumor immune microenvironment, increasing CD8^+^ T cell infiltration	Getz et al., 2021; Kansal et al., 2023
MitoTam	UT-SCC-5	Increases radiosensitivity of RT-resistant HNSCC cells	Induces mitochondrial lipid peroxidation, inhibits the antioxidant defense system of RT-resistant cells, and promotes ferroptosis by disrupting mitochondrial GPX4 function	Reinema et al. (2024)
Artemisinin derivatives (e.g., artesunate, dihydroartemisinin)	HN3-cisR, HN4-cisR, HN9-cisR KYSE150, KYSE30	Triggers ferroptosis in HNSCC cell Reverses ESCC multidrug resistance	Inhibits AKT/mTOR pathway and downregulate GPX4, thereby inducing ferroptosis Triggers ferritinophagy to release labile Fe^2+^ to enhance Fenton reaction	Roh et al., 2017; Xia et al., 2024b; Chen et al., 2020
TMEM44-AS1	KYSE30, KYSE150,ECa109	Reduces the sensitivity of ESCC cells to ferroptosis *in vitro* and *in vivo*	Upregulates GPX4 expression by the IGF2BP2-GPX4 axis	Yang et al. (2023d)
Cuproptosis				
Elesclomol	T47D, K562, HeLa, NCIH441, HepG2, Hep3B, Huh7, etc.	Induces cuproptosis in cancer cells	Activates FDX1, promoting Cu^2+^ reduction to Cu^+^ and subsequent lipoylated protein aggregation Inhibits the PI3K/mTOR pathway in HCC.	Tsvetkov et al. (2019); Li et al., 2023e
Copper tetrathiol molybdate (TM)	SAS, HSC-3, HSC-4	Reverses cisplatin resistance in HNSCC.	Downregulating ATP7B expression to increase intracellular copper accumulation, enhancing cisplatin-induced cuproptosis	Ogawa et al. (2024)
Ferroptosis + cuproptosis				
Sorafenib	UT-SCC 42B, Huh7	Enhances radiotherapy sensitivity in HNSCC cells Synergizes with artesunate to induce ferroptosis in HCC cells	Inhibits system Xc^−^ to reduce GSH synthesis Downregulates FDX1 degradation, indirectly promoting cuproptosis while inducing ferroptosis	Beizaei et al. (2019); Wang et al. (2024)
DSF/Cu (Disulfiram/Copper)	FaDu, Hep2, HCC-LM3 5-8F, CNE2, S18, 6-10B; Huh7, Hepa1-6, SMMC-7721, etc.	Enhances ferroptosis/cuproptosis and promotes autophagy-mediated cell death Induces apoptosis and necrosis of NPC cells and CAF through ROS-induced ferroptosis *in vitro* and *in vivo* Enhances the therapeutic efficacy in glioblastoma patients Enhances anti-tumor immunity in HCC cells	Forms CuET to deplete GSH for ferroptosis; promotes mitochondrial Cu^2+^ accumulation for cuproptosis Induces ferroptosis in NPC cells via the ROS/MAPK-p53 pathway Activates DCs and upregulates macrophage function in HCC.	Park et al. (2018); Huang et al., 2024b; Li et al., 2024a; Zhang et al., 2024c; Gao et al., 2022

### 4.4 Nanotherapeutics

Cancer therapies targeting ferroptosis and cuproptosis are current research hotspots, but they still face numerous challenges. The core regulatory molecules of these two cell death pathways (such as iron and copper metabolism-related proteins) also play crucial roles in normal cells, rendering the development of tumor-selective drugs particularly challenging. Tumor cells can develop drug resistance through multiple mechanisms, including upregulating antioxidant systems (e.g., GPX4), altering metal ion metabolism, or activating alternative survival pathways. The microenvironmental characteristics of different tumor types (such as hypoxia levels, nutrient availability, and immune infiltration) also significantly impact therapeutic outcomes. Furthermore, systemic intervention in iron/copper metabolism may induce systemic side effects like anemia and neurotoxicity, necessitating precise control of the therapeutic scope by treatment regimens. Therefore, achieving the specific accumulation of drugs at tumor sites remains a key challenge, which necessitates the development of novel delivery systems such as responsive nanocarriers and antibody-drug conjugates.

#### 4.4.1 Nanomaterials as drug delivery vehicles

Iron and copper primarily exist in compound form in nature; however, when incorporated into substrates, they can impart unique properties, such as adjustable porosity, mechanical strength, degradability, and framework cross-linking capabilities. In recent years, iron-based and copper-based nanomaterials have emerged as novel drug delivery systems, demonstrating distinct advantages in tumor-targeted drug therapy due to their multifunctional characteristics. On one hand, through surface modifications (e.g., hyaluronic acid coating) or responsiveness to tumor microenvironment features (acidic pH, high GSH, etc.), they enable tumor-specific targeting and precise delivery, significantly enhancing drug accumulation in tumor tissues. On the other hand, these carriers can directly supplement death effector molecules (Fe^2+^/Cu^2+^) or induce cell death by interacting with ferroptosis and cuproptosis inducers, thereby synergistically inhibiting tumor growth and markedly improving the therapeutic efficacy of conventional drugs. In addition, nanoparticles can protect drugs from degradation, enhance their stability, and prolong their half-life.

For example, iron-based MOF materials interact with abundant GSH in the tumor microenvironment, causing structural disintegration and releasing Fe^2+^/Fe^3+^ ions. These ions synergize with the large amount of H_2_O_2_ generated by GOx catalysis to trigger a potent Fenton reaction, significantly increasing hydroxyl radical levels (Wan et al., 2020). The acid-responsive iron-based nanocomposite UPM (USPBNPs@MCSNs) exhibits dual enzymatic activity in weakly acidic environments, generating toxic ·OH to kill tumor cells, while releasing O_2_ in neutral environments to alleviate hypoxia. Additionally, it downregulates the xCT/GPX4/GSH axis to induce ferroptosis, achieving favorable therapeutic outcomes in the treatment of OSCC in mice (Zhao et al., 2024). Currently, the design of such nanomaterials has evolved from single-functionality toward intelligent, multi-modal synergy. Wang et al. encapsulated the chemotherapeutic drug doxorubicin (DOX) into mesoporous silica nanoparticles, and further modified the surface with a pH-sensitive metal polyphenol coating (consisting of iron ions and epigallocatechin gallate) to develop a synergistic therapeutic system combining chemotherapy and ferroptosis—DMEFe nanoparticles (NPs)—for the treatment of OSCC. This nanoparticle system shows excellent pH-responsive controlled drug release performance and can be efficiently taken up by the OSCC cell line SSC-25, thereby significantly suppressing the proliferation of these cells. By upregulating ROS and regulating ferroptosis-related genes, it achieves chemo-ferroptosis synergistic therapy, thus providing a new strategy for OSCC (Wang et al., 2024b). The CuO(2)@G5-BS/TF nanocomplex can target tumor cells overexpressing carbonic anhydrase IX (PC@B-HCAIX) through specific binding with p-carboxybenzenesulfonamide (BS), inhibit their CAIX activity, thereby reducing intracellular pH to accelerate the release of Fe^3+^/Cu^2+^, self-supply of H_2_O_2_, and Fenton reaction. Furthermore, it can impede tumor metastasis by mitigating the extracellular acidity within the TME. The reduction of Fe^3+^/Cu^2+^ by intracellular GSH can further amplify ROS levels, while the depletion of GSH in turn inhibits GPX-4-mediated antioxidant responses to induce ferroptosis, thereby enhancing therapeutic efficacy (Huang H. et al., 2024). Meng et al. developed a multifunctional nanoplatform CCDRH (CeO_2_@CuO_2_@DOX-RSL3@HA) that achieves potent antitumor efficacy through multiple mechanisms of action. Within the acidic TME, CuO_2_ decomposes to generate Cu^2+^ and H_2_O_2_; the latter is then catalytically converted by CeO_2_ into ·OH and O_2_, thereby enhancing chemodynamic therapy. RSL3 inhibits GPX4 expression, while Cu^2+^ and Ce^4+^ deplete GSH, synergistically inducing ferroptosis. Concurrently, Cu^+^ triggers cuproptosis by promoting DLAT oligomerization and downregulating Fe-S cluster proteins, demonstrating high performance in tumor suppression (Meng et al., 2025). Copper-iron bimetallic sulfide nanoparticles (CFS NPs), synthesized through a valence-regulated metal ion strategy, enable the controlled release of Cu^+^ and Fe^2+^, effectively catalyzing Fenton-like reactions. Meanwhile, Fe^3+^ maintains Fe^2+^ cycling and consumes GSH, thereby amplifying mitochondrial oxidative stress and synergistically inducing cell apoptosis, cuproptosis, and ferroptosis. By precisely regulating metal valence states and spatial distribution, CFS NPs overcome the randomness limitation of traditional metal ion interference therapy (MIIT), significantly enhance tumor oxidative damage, and achieve efficient tumor suppression. Furthermore, owing to the GSH buffering effect in normal cells, they exhibit high biosafety, offering a novel strategy for multimodal combined tumor therapy (Yu et al., 2025).

While these metal ion-based nanomaterials achieve efficient anti-tumor effects, significant breakthroughs have also been made in the intelligent design of their delivery systems. From traditional pH-responsive materials to current biomimetic delivery systems based on extracellular exosomes, researchers have continuously optimized the targeting capability and biocompatibility of nanocarriers. For example, NK cell-derived exosome-encapsulated AuMn nanoclusters (Exo-AMNCs) not only retain the OSCC-targeted ferroptosis-inducing ability but also exhibit active tumor targeting and real-time imaging functions, marking a crucial advancement in nanomedicine for HNSCC treatment (Yang et al., 2025). Other nanomaterials can also exert anti-tumor effects by inducing ferroptosis or cuproptosis through multiple pathways. For instance, TDN@EVs (tetrahedral DNA nanostructure-modified M1-EVs) trigger ferroptosis, mitochondrial stress, and DNA damage via Hsc70-mediated GPX4 degradation (Wang D. et al., 2025). The therapeutic nanoplatform Zn@CDDP@HMON, based on hollow mesoporous manganese dioxide nanoparticles (HMON), releases Zn (2+) and Pt (2+) ions in the acidic tumor microenvironment of OSCC. These ions inhibit mitochondrial respiration and activate NADPH oxidase (NOX), thereby increasing the production of superoxide anions and hydrogen peroxide. The released Mn (4+) can also deplete intracellular GSH to stimulate Fenton-like reactions, sensitizing tumor cells to ferroptosis (Chang et al., 2025). Various traditional Chinese medicine components can also be delivered. In recent years, the anticancer effect of the traditional Chinese medicine monomer artesunate (ART) has attracted widespread attention in the treatment of ESCC owing to its characteristics of low toxicity, high efficiency, and ability to reverse multidrug resistance (MDR). To overcome the poor water solubility and bioavailability of ART, Xia et al. developed artesunate-loaded solid lipid nanoparticles (SLNART). This formulation upregulates TFR to increase Fe^2+^ ions and inhibits the AKT/mTOR signaling pathway to downregulate GPX4, thereby inducing tumor cell ferroptosis (Xia Y. et al., 2024). These studies, through the innovation and optimization of nanocarriers, have not only significantly improved the delivery efficiency of traditional chemotherapeutic drugs and overcome tumor drug resistance, but also exhibited unique multifunctional synergistic effects in tumor therapy, thereby providing innovative strategies for the development of highly effective and low-toxic HNSCC nanotherapies.

#### 4.4.2 Nanomaterial-based combination therapy

In recent years, nanotherapies targeting ferroptosis and cuproptosis have also been combined to provide innovative synergistic strategies for enhancing tumor radiotherapy and immunotherapy. Nanoassemblies are prepared by leveraging the affinity between fluorophenylboronic acid-modified kidney-clearable ultrasmall iron nanoparticles (USINPs) and (131) I-aPD-L1, enabling effective tumor targeting and decomposition in the presence of ATP within the tumor microenvironment. Both *in vitro* and *in vivo* studies confirm that USINP-induced ferroptosis increases tumor radiosensitivity, which is further amplified by (131) I-mediated radionuclide therapy (RPT). Moreover, the combined action of RPT-induced radiolysis, ferroptosis-mediated lipid peroxidation, and PD-L1 blockade synergistically induces immunogenic cell death, generating potent anti-tumor immune responses (Shen et al., 2025). Wang et al. developed cell membrane-encapsulated semiconductor polymer nanoparticles for co-delivery of circADARB1-targeting siRNA and iron ions. This nanoplatform demonstrated effective suppression of circADARB1 expression with concurrent iron accumulation, and synergistic enhancement of radiotherapy efficacy through promoted ferroptosis and increased radiosensitivity in NPC cells (Wang D. et al., 2024). Studies have revealed that the cuproptosis regulators FDX1 and LIAS are upregulated in residual tumors after radiotherapy, thereby increasing sensitivity to cuproptosis. Copper-containing nanocapsule-like polyoxometalates have been employed to release copper ions in a controlled manner upon exposure to ionizing radiation. At clinically relevant radiation doses, radiation-triggered cuproptosis helps overcome acquired radiation resistance and activates a robust abscopal effect, achieving a cure rate of up to 40% in radioresistant and re-irradiated tumor models (Liao et al., 2024). The novel PVP-modified copper/hafnium-doped phosphate nanostimulator (CHP) enables cuproptosis-synergized radiosensitization under low-dose X-ray radiation and activates immunogenic cell death (ICD) to enhance anti-tumor immunity by depleting GSH, alleviating hypoxia, and reprogramming the tumor microenvironment (Jiang X. et al., 2024). The functional nanozyme system CussOMEp integrates copper-based nanovectors (CussNV) with the copper transporter inhibitor OME. The system achieves dual therapeutic effects by directly inducing synergistic ferroptosis and cuproptosis through hydroxyl radical generation and ATP7A copper transporter inhibition while modulating the tumor microenvironment to enhance immunocyte activation, triggering a robust immunogenic antitumor response. When used in combination with αPD-1 therapy, CussOMEp exhibits significant antitumor effects (Gu et al., 2025).

Many traditional Chinese medicines have shown great potential in the combined treatment of tumors. The nanocomplex PC@B-H, fabricated by Sun et al., utilizes the acidic and reductive properties of the OSCC tumor microenvironment to release copper ions and Plumbagin. This not only triggers ferroptosis and cuproptosis but also promotes DC maturation and CTL infiltration, thereby providing long-lasting anti-tumor immunity (Sun et al., 2025). The novel multifunctional copper-based nanocomposite RCL@Pd@CuZ integrates multiple functionalities, including RGD-targeted modification, capsaicin-mediated hypoxia alleviation, ROS generation by Cu MOFs, cuproptosis induction via Cu^2+^ release, oxygen production catalyzed by Pd nanozymes, and enhanced X-ray absorption. These integrated functions enable it to simultaneously improve the tumor microenvironment, amplify oxidative stress, and promote immunogenic cell death. Animal experiments have confirmed that when combined with radiotherapy, this nanoplatform can significantly inhibit tumor growth (>90%), promote DC maturation and CD8^+^ T cell infiltration, thereby providing a novel cuproptosis-related radioimmunotherapy strategy to overcome radioresistance (Li R. et al., 2024). Zhu et al. developed a novel nanotheranostic platform (177Lu-MFeCu@Tan) by co-loading the radionuclide 177Lu and tanshinone into Fe/Cu bimetallic nanozymes, which enables SPECT imaging-guided quadruple synergistic therapy. Experiments have verified that this strategy significantly enhances tumor killing efficacy and inhibits recurrence by simultaneously overcoming radiotherapy/chemotherapy resistance (reducing H2O2 and GSH levels), inducing mitochondrial dysfunction (ROS accumulation), and activating cuproptosis (DLAT oligomerization and lipid peroxidation) (Zhu et al., 2025). Metal ion-based multifunctional nanomaterials demonstrate significant potential for “theranostics integration” through precise modulation of the tumor microenvironment, enhancement of radiosensitivity, and activation of immune responses.

#### 4.4.3 Nano-enabled phototherapy

In recent years, photothermal therapy (PTT) and photodynamic therapy (PDT), as emerging non-invasive tumor treatment modalities, have demonstrated significant value in HNSCC treatment owing to their advantages of precise targeting and low toxic side effects. PTT employs near-infrared light (700–1,100 nm) to excite photothermal materials, generating local hyperthermia to kill tumors, while PDT relies on photosensitizers that, upon excitation by specific wavelengths of light, produce ROS to exert cytotoxic effects (Du et al., 2022). Studies have indicated that these two phototherapeutic approaches not only directly eliminate tumors but also enhance regulating cell death mechanisms including ferroptosis and cuproptosis. For instance, a study conducted by Shi et al. showed that the utilization of copper cysteamine as a photosensitizer in X-ray-activated photodynamic therapy (X-PDT) can significantly suppress the growth of squamous cell carcinoma (Shi L. et al., 2019). Wang et al. developed near-infrared (NIR) light-controlled nanoparticles (NPs), CuD@PM, which can selectively deliver copper to HNSCC cells and induce copper deposition when microneedles loaded with triacetylated azacitidine (TAc-AzaC) are present and 808 nm laser irradiation is applied. Intravenous administration of these NPs markedly suppressed tumor progression and potentiated the anti-tumor immune response in HNSCC animal models (Wang et al., 2024c). Zhang et al. developed an injectable self-healing CHPP hydrogel (CMCS/HA-CHO/PVP-CuO2/PDA). This intelligent hydrogel enables responsive release of H2O2 in the tumor microenvironment by specifically targeting the CD44 receptor on the surface of tumor cells. Subsequently, Cu^2+^ catalyzes the generation of highly toxic hydroxyl radicals (·OH) while continuously depleting intracellular GSH, synergistically inducing ferroptosis and cuproptosis in tumor cells to enhance the anti-tumor efficacy of PTT. This combined therapy exhibits significant killing efficacy against OSCC (Zhang X. et al., 2025). These studies not only confirm the critical role of PTT/PDT in HNSCC treatment but also reveal the vast potential of combining phototherapy with emerging cell death mechanisms (ferroptosis/cuproptosis), thereby providing valuable insights for developing efficient and low-toxicity HNSCC therapeutic strategies.

As shown in Table 3, the unique properties of nanomaterials have led to the rapid development of metal-based cell death pathways in HNSCC treatment. However, the development and production of nanoparticles are complex and expensive, with large-scale manufacturing and standardization remaining significant hurdles. Furthermore, controlling the *in vivo* biodistribution and clearance of nanoparticles presents considerable challenges. Despite the promising results observed in current laboratory studies, the translation of these findings into clinical applications necessitates rigorous pharmacodynamic evaluation and safety validation. It is anticipated that with the continuous advancement of research into the interaction mechanisms between ferroptosis and cuproptosis, alongside the development of intelligent responsive nanocarriers, nanotherapies targeting metal death pathways will emerge as a key breakthrough for improving the prognosis of HNSCC.

**TABLE 3:** Comprehensive nanotherapies targeting ferroptosis, cuproptosis, and combinations for HNSCC treatment.

Nanotherapy type	Cell lines	Regulated cell death type	Mechanism of action	Therapeutic efficacy in HNSCC	References
Acid-responsive iron-based nanocomposite (USPBNPs@MCSNs, UPM)	CAL27, HN6	Ferroptosis	Releases Fe^2+^ in acidic OSCC TME to trigger Fenton reaction (·OH) Downregulates xCT/GPX4/GSH axis Releases O_2_ to alleviate hypoxia	Inhibits OSCC tumor growth in mice	Zhao et al. (2024)
Mitochondrial-targeted tamoxifen analog nanosystem (MitoTam-modified Fe_3_O_4_ NPs)	UT-SCC-40,UT-SCC-5	Ferroptosis	Targets HNSCC mitochondria Inhibits antioxidant system in radioresistant cells Disrupts mitochondrial GPX4 function	Enhances radiosensitivity of RT-resistant HNSCC Shrinks xenografts, prolongs survival	Reinema et al. (2024)
NK cell-derived exosomes + AuMn nanoclusters (Exo-AMNCs)	SCC9,CAL27, CRL1623,NK92,HEK293T, NCM460,GES-1	Ferroptosis	Achieves active targeting to OSCC via NK exosomes Releases AuMn to induce Fe^2+^-dependent lipid peroxidation Enables real-time imaging	Ferroptosis-induced suppression of OSCC proliferation and metastasis, with low systemic toxicity	Yang et al. (2025)
Tetrahedral DNA nanostructure-modified M1 macrophage EVs (TDN@EVs)	RAW 264.7,SCC7,HOK	Ferroptosis	Delivers TDN to OSCC Triggers Hsc70-mediated GPX4 degradation Induces mitochondrial stress and DNA damage	Enhances OSCC therapy efficacy	Wang et al. (2025a)
Artesunate-loaded solid lipid nanoparticles (SLNART)	KYSE150,KYSE30,HEEC	Ferroptosis	Delivers artesunate to ESCC Upregulates TFR (Fe^2+^ influx) Inhibits AKT/mTOR, downregulates GPX4	Induces ferroptosis in ESCC cells Reverses ESCC multidrug resistance	Xia et al. (2024b)
Hollow mesoporous MnO_2_ NPs (Zn@CDDP@HMON)	CAL-27	Ferroptosis + chemotherapy	Releases Zn^2+^/Pt^2+^/Mn^4+^ in acidic OSCC TME Inhibits mitochondrial respiration, activates NOX (ROS) Mn^4+^ depletes GSH, sensitizes to ferroptosis	Enhances ferroptosis sensitivity of OSCC.	Chang et al. (2025)
Doxorubicin-loaded mesoporous silica NPs (DMEFe NPs: Fe^3+^-epigallocatechin gallate coating)	SSC-25	Ferroptosis + chemotherapy	Enables pH-responsive release of DOX and Fe^3+^ (Fe^3+^ is reduced to Fe^2+^) Upregulates ROS and modulates ferroptosis-related genes	Achieves chemo-ferroptosis synergy in OSCC cells	Wang et al. (2024a)
NIR-activated copper nanoplatform (CuD@PM + TAc-AzaC microneedles)	4MOSC1	Cuproptosis	Selectively delivers copper to HNSCC cells and induces cuproptosis	Suppresses HNSCC progression in animals	Wang et al. (2024c)
Copper-based nanocapsular polyoxometalates	BEAS-2B, HaCaT, A549, HCT116, Hep-1-6, HUVEC, etc.	Cuproptosis + radiosensitization	Ionizing radiation triggers Cu^2+^ release	Overcomes HNSCC radioresistance Achieves 40% cure rate in RT-resistant models	Liao et al. (2024)
Injectable self-healing CHPP hydrogel (CMCS/HA-CHO/PVP-CuO_2_/PDA)	CAL-27, HSC-3	Cuproptosis + ferroptosis + photothermal therapy (PTT)	Targets CD44^+^ HNSCC, releases H_2_O_2_/Cu^2+^ Cu^2+^ catalyzes ·OH, depletes GSH.	Kills OSCC cells via synergistic PTT/chemodynamic therapy	Zhang et al. (2025c)
CuO(2)@G5-BS/TF nanocomplex	4T1	Ferroptosis + cuproptosis	Targets CAIX^+^ HNSCC via BS Releases Fe^3+^/Cu^2+^ (GSH-reduced to Fe^2+^/Cu^+^) -Fenton reaction (·OH) + Cu^+^-induced DLAT oligomerization	Reduces extracellular acidity (blocks metastasis) Enhances ferroptosis/cuproptosis in OSCC.	Huang et al. (2024a)
Copper-iron bimetallic sulfide nanozymes (CFS NPs)	4T1, L929, HepG2	Ferroptosis + cuproptosis	Controlled release of Cu^+^ and Fe^2+^ Fe^3+^ cycles to consume GSH Avoids GSH depletion in normal cells	Achieves >85% tumor inhibition in HNSCC models with no obvious oral mucosal damage	Yu et al. (2025)
TME-responsive nanoplatform (CCDRH: CeO_2_@CuO_2_@DOX-RSL3@HA)	4T1	Ferroptosis + cuproptosis + chemotherapy	CuO_2_ decomposes to Cu^2+^ (cuproptosis) and H_2_O_2_ CeO_2_ catalyzes ·OH (ferroptosis), RSL3 inhibits GPX4 - HA targets CD44^+^ HNSCC.	Synergistically induces dual cell death in HNSCC, effective suppress tumor	Meng et al. (2025)
PVP-modified Cu-Hf-doped phosphate nanostimulators (CHP)	4T1	Ferroptosis + cuproptosis + radiosensitization	Low-dose X-rays trigger Cu^2+^ release (cuproptosis) and Hf enhances radiation absorption Depletes GSH (inhibits GPX4) Activates immunogenic cell death (ICD)	Achieves >90% tumor inhibition when combined with radiotherapy Improves immunosuppressive TME.	Jiang et al. (2024b)
Copper-based cascade nanocomplexes (PC@B-H)	MOC-1	Ferroptosis + cuproptosis + immunomodulation	Releases Cu^2+^ and plumbagin in OSCC TME Triggers ferroptosis/cuproptosis Promotes DC maturation and CTL infiltration	Induces long-term anti-tumor immunity Inhibits OSCC growth and recurrence	Sun et al. (2025)
Multifunctional copper-based nanocomposite (RCL@Pd@CuZ)	MC38	Ferroptosis + cuproptosis + radiotherapy	RGD targets HNSCC Pd catalyzes O_2_ production (alleviates hypoxia), CuZ releases Cu^2+^ (cuproptosis) Enhances X-ray absorption, triggers ferroptosis	Achieves >90% tumor inhibition with radiotherapy Promotes DC/CD8^+^ T cell activation	Li et al. (2024c)
Functional nanozyme system (CussOMEp: Cu-based nanocarriers + OME)	4T1,Luc-4T1	Ferroptosis + cuproptosis + immunomodulation	Cu-based nanocarriers generate ·OH (ferroptosis); OME inhibits ATP7A (copper accumulation) Modulates TME (enhances DC/CD8^+^ T cell activity)	Synergizes with αPD-1 resulted in significantly smaller primary tumors Exhibited robust inhibition of mice distant tumor growth Activates systemic anti-tumor immunity	Gu et al. (2025)
Tanshinone-loaded Fe/Cu bimetallic nanozymes (177Lu-MFeCu@Tan)	4T1	Ferroptosis + cuproptosis + radiotherapy	177Lu mediates radiotherapy Generate highly active free radicals Chemotherapy via tanshinones release Glutathione depletion promotes ferroptosis and cuproptosis via enhanced lipid peroxidation	Quadruple synergy (RT + dual cell death + immunity) Reduces HNSCC recurrence	Zhu et al. (2025)

Ferroptosis and cuproptosis represent a highly promising yet still nascent field in the treatment of HNSCC. Currently, a phase I clinical trial investigating intratumoral injection of carbon nanosphere-iron [CNSI-Fe (II)] for advanced solid tumors has been completed (6 February 2025), demonstrating favorable safety and tolerability profiles (NCT06048367). Cisplatin plus cetuximab (CX) is one of the standarded first-line treatments for HNSCC. However, this therapeutic regimen is often associated with high toxicity and drug resistance. Valproic acid (VPA) enhances tumor suppression by reducing mRNA expression of ERCC excision repair 1, as well as increasing intracellular cisplatin concentration through upregulating the cisplatin influx channel CTR1 and downregulating the cisplatin efflux transporter ATPase ATP7B at the transcriptional level (Iannelli et al., 2020). Currently, clinical trials exploring the combination therapy of VPA and CDDP/CX in patients with R/M HNSCC have advanced to phase II (NCT02624128). Although existing studies are primarily preclinical mechanistic explorations, they have laid an important theoretical foundation for the development of novel targeted and combination therapeutic strategies for HNSCC.

## 5 Challenges of targeting metal ion homeostasis in HNSCC patients

### 5.1 Limitations of current research

Current studies on metal ion homeostasis in HNSCC mostly rely on cell lines, lacking further animal experiments and clinical validation. In addition, multiple molecules have been studied individually, but the Interaction network between them remains unclear, such as how the FTX/FEN1/ACSL4 axis and miRNA synergistically regulate. HNSCC includes different subgroups, different primary sites (oral cavity, Larynx, Pharynx, etc.) and different driver gene backgrounds. Existing studies rarely distinguish these backgrounds. For example, ACSL4 seems to play different roles in OSCC and ESCC (Wang X. et al., 2024; Xia L. et al., 2024), but the underlying reasons have not been deeply explored. It is questionable whether a certain target is universally effective in all HNSCC patients. Currently, research on the association between cuproptosis and HNSCC is also insufficient, mostly derived from models of other cancer types (such as Hepatocellular carcinoma of liver, Neuroblastoma), and the universality of these findings in HNSCC requires further direct verification.

### 5.2 Tumor heterogeneity

Although targeting ferroptosis and cuproptosis presents a promising therapeutic avenue for HNSCC, accumulating evidence underscores significant inter-patient heterogeneity in treatment response, largely dictated by HPV status, key genetic alterations, and the TME. Firstly, HPV status serves as a fundamental stratifying factor. For instance, statins, acting as ferroptosis inducers, demonstrate a more pronounced protective effect in HPV-positive patients, whereas the ferroptosis-PD-L1 regulatory axis is more active in HPV-negative tumors, suggesting that ferroptosis signatures may predict immunotherapy response in this subgroup (Getz et al., 2021; Chung et al., 2023). Secondly, specific genetic mutational profiles further refine responsive populations. The high frequency of CDKN2A mutations in HPV-negative HNSCC and TP53 mutations co-occurring with HPV that lead to upregulated AURKA expression define subgroups with distinct therapeutic vulnerabilities (e.g., sensitivity to mevalonate pathway inhibition) and resistances (e.g., cisplatin resistance reversible by AURKA inhibitors) (Jiang et al., 2022; Jia et al., 2024). Furthermore, the TME, particularly the heterogeneity of CAFs, is a critical source of resistance. Studies have confirmed that PDPN + CAFs can directly suppress ferroptosis via specific signaling axes to enhance tumor aggressiveness, while CAF-derived exosomes can modulate copper homeostasis by targeting the copper transporter ATP7A, thereby promoting cancer progression (Li et al., 2023d; Zhang S. et al., 2025). In conclusion, the future development of ferroptosis/cuproptosis-targeting therapies must adopt a biomarker-driven precision medicine strategy: exploring statin-based combinations for HPV-positive patients; employing corresponding targeted agents (e.g., AURKA inhibitors) for tumors harboring specific mutations (e.g., TP53 mutation/CDKN2A loss) to overcome resistance; and developing therapies that target specific CAF subpopulations or their signaling pathways to counteract the microenvironmental suppression of cell death, ultimately maximizing therapeutic efficacy on an individualized basis.

### 5.3 Potential systemic toxicity

Moreover, due to the high sensitivity of vital organs such as the liver and brain to metal ions, disrupting iron or copper homeostasis may damage healthy tissues while inhibiting tumor cells. Studies have reported that GPX4 inhibitors, as key mediators regulating ferroptosis, can adversely affect the development and function of the nervous system and kidneys (Liang et al., 2019; Koppula et al., 2021). Long-term targeting of iron or copper metabolism may induce iron overload or copper toxicity, causing long-term damage to multiple tissues and organs (Kciuk et al., 2024; Zhou et al., 2024), thereby posing a long-term health threat to patients. Against this background of potential systemic toxicity, monitoring indicators closely linked to the core mechanisms of ferroptosis/cuproptosis and metal ion metabolism become particularly critical. In addition to serum copper/iron levels, which directly reflect systemic metal ion balance, ceruloplasmin—an essential protein regulating copper transport and homeostasis—also indirectly reflects systemic copper metabolism status (Mzhel’skaya, 2000). Furthermore, as a crosstalk node between ferroptosis and cuproptosis, GSH status serves as a central indicator of cellular antioxidant capacity. Based on these mechanistic associations, these indicators are speculated to serve as potential safety monitoring targets for combined ferroptosis/cuproptosis-targeted therapies in HNSCC. However, their clinical applicability as standardized monitoring indices, along with the establishment of appropriate reference ranges for HNSCC patients, remain to be validated through well-designed prospective clinical studies. Therefore, developing combination therapies with high target specificity is an arduous task. It is difficult to design strategies that can selectively induce ferroptosis and cuproptosis in HNSCC cells while protecting adjacent healthy tissues. The lack of such targeting increases the risk of widespread cytotoxicity, thereby limiting the clinical application of combination therapies.

So inducing ferroptosis and cuproptosis in HNSCC now still represents several challenges. First, the current knowledge about the *in vivo* regulatory mechanisms of ferroptosis and cuproptosis pathways in HNSCC remains incomplete. This knowledge gap may cause unexpected toxicities when the combined therapy is applied in clinical settings. It also increases the risk of unforeseen interactions between the combined therapy and other cell death mechanisms (e.g., apoptosis, autophagy), and such interactions will bring uncertainties to the therapeutic outcomes. Meanwhile, the tumor heterogeneity of HNSCC increases the complexity of combination therapy. Different cancer cell subpopulations exhibit substantial differences in sensitivity to ferroptosis and cuproptosis. Some drug-resistant subpopulations can evade immune cell death or acquire therapeutic resistance by enhancing antioxidant defense systems or regulating intracellular metal metabolism, ultimately leading to incomplete tumor elimination. Additionally, the therapy is also able to trigger inflammatory reactions. Specifically, lipid peroxidation linked to ferroptosis and mitochondrial dysfunction associated with cuproptosis both have the potential to amplify inflammatory cascades. Such amplification will disrupt the local tumor microenvironment, and it may also promote the progression of HNSCC as well as the emergence of treatment resistance.

## 6 Summary and discussion

Metal ions play crucial roles in numerous physiological processes, including signal transduction, energy metabolism, and oxidative stress regulation. Given their involvement in tumorigenesis and cancer progression, targeting metal ion metabolism has emerged as a promising anti-tumor strategy. Notably, both iron and copper possess high redox potential, enabling them to participate in Fenton reactions and generate ROS, which contribute to their cytotoxic effects in tumor cells. Although iron is currently regarded as the sole metal ion triggering ferroptosis, accumulating evidence indicates that ferroptosis represents a metal-dependent cell death modality involving both iron and copper (Li et al., 2023c). Despite cuproptosis was discovered relatively recently, growing evidence highlights its significant therapeutic potential in cancer treatment. Furthermore, cuproptosis and ferroptosis are closely and complexly interconnected through various ions and pathways involved in mitochondrial energy metabolism, oxidative stress, GSH metabolism, and the induction and progression of autophagy.

Beyond prognostic analysis, the exploration of a personalized therapeutic algorithm framework for HNSCC based on FRG/CRG also represents a potential future research direction. For example, patients may be initially stratified by HPV/p16 status, subsequently complemented by core FRG/CRG profiling. Risk scoring guides therapeutic decisions (standard therapy for low-risk, synergistic ferroptosis/cuproptosis-targeted therapy for high-risk), with dynamic optimization via periodic biomarker revalidation and panel expansion to achieve precision diagnosis and treatment.

Preliminary studies have explored the combination of these two approaches for anti-tumor therapy. For example, sorafenib and erastin can simultaneously promote copper-dependent protein lipoylation and ferroptosis in tumor cells by inhibiting FDX1 degradation and GSH synthesis (Wang W. et al., 2023). Additionally, recent research indicates that the classic drug DSF can activate both ferroptosis and cuproptosis, offering a “dual-pathway” anti-tumor strategy. Its metabolite DTC forms a CuET complex with copper. On one hand, this complex inhibits GPX4 activity by depleting GSH, causing the accumulation of lipid peroxidation products and triggering ferroptosis; On the other hand, acting as a copper ionophore, it facilitates mitochondrial copper accumulation, disrupts the TCA cycle, and induces abnormal aggregation of lipoylated proteins (such as DLAT), thereby triggering cuproptosis. These two death pathways collectively exacerbate ROS bursts, with particularly significant effects on tumors dependent on antioxidant defense mechanisms (such as high SLC7A11 expression or KRAS mutations) (Li H. et al., 2024). Preclinical studies have shown that combining DSF with copper salts or ferroptosis inducers enhances therapeutic efficacy, and clinical trials (e.g., for glioblastoma) have preliminarily validated this potential (Huang J. et al., 2024). In NPC, the DSF-Cu complex triggers ferroptosis through ROS-mediated activation of the MAPK-p53 signaling pathway. When combined with cisplatin, it synergistically inhibits tumor growth with good *in vivo* tolerability (Li et al., 2020). To advance the translational application of this drug repurposing strategy in HNSCC, future efforts should focus on optimizing targeted delivery and screening sensitive patient populations. This also highlights that additional drug mechanisms related to programmed cell death warrant further exploration.

In addition to the synergistic induction of ferroptosis and cuproptosis, exploring combination strategies involving PCD pathways with existing anti-tumor therapies and emerging therapeutic technologies may provide new insights to overcome current bottlenecks in cancer treatment. PD-1/PD-L1 inhibitors have been utilized in numerous tumor studies to promote ferroptosis in cancer cells, while copper ions have also been linked to PD-L1 expression at both transcriptional and protein levels in malignant tumors (Yang F. et al., 2023; Zhang et al., 2024b). However, there is still a lack of relevant research on targeting the combination of metal-related PCD and immune checkpoint inhibitors in HNSCC, which may be a novel strategy to enhance the therapeutic efficacy of HNSCC treatment. Research into the application of nanomaterials for tumor therapy has also advanced rapidly in recent years. Nanomaterials enable precise tumor targeting, significantly enhancing local drug accumulation. The delivery of metal ions into cells via nanomaterials constitutes an alternative strategy to induce ferroptosis and cuproptosis. Although there are still many problems at present, iron- and copper-based nanomaterials demonstrate considerable therapeutic potential, offering novel treatment approaches for HNSCC.

Targeting metal ion homeostasis to induce ferroptosis and cuproptosis offers a highly promising therapeutic direction for HNSCC, but it still faces multiple core challenges to date. Current studies mainly rely on cell line models, lacking sufficient *in vivo* experiments and clinical validation, and the synergistic regulatory network of molecules related to metal ion metabolism remains unclear. The high heterogeneity of HNSCC, including differences in HPV status, primary tumor sites, driver gene mutation profiles, and tumor microenvironment heterogeneity, leads to significant inter-individual variations in treatment response, making the traditional “one-size-fits-all” therapeutic model ineffective. Meanwhile, potential systemic toxicity and treatment-related inflammatory reactions induced by targeted therapies further limit their clinical translation. Therefore, future research should focus on several indispensable directions: clarifying the specific *in vivo* regulatory mechanisms of metal ion homeostasis, ferroptosis, and cuproptosis in HNSCC; identifying precise biomarkers for patient stratification; standardizing the clinical reference ranges of toxicity monitoring indicators; developing combined therapeutic strategies targeting the tumor microenvironment; and regulating treatment-related inflammatory responses to avoid drug resistance and tumor progression. The resolution of these issues will lay the foundation for transforming ferroptosis/cuproptosis-targeted therapies toward a biomarker-driven precision medicine paradigm, ultimately achieving the synergistic improvement of therapeutic efficacy and safety in HNSCC patients.
