# Supplementary material for: Ferroptosis and cuproptosis in head and neck squamous cell carcinoma: interconnected mechanisms and therapeutic implications
Source: Front Pharmacol. 2026 Feb 13;17:1694895. doi: 10.3389/fphar.2026.1694895 (PMC12946670; doi:10.3389/fphar.2026.1694895)
Supplement: Supplementary file 1 [file Supplementaryfile1.docx]

**Supplementary material 1**

## Mechanisms and crosstalk between ferroptosis and cuproptosis in HNSCC

### 1. Specific mechanisms of ferroptosis

Iron is indispensable for key biological processes, such as hemoglobin synthesis, mitochondrial electron transport in the respiratory chain, and cell cycle regulation. However, disruptions in iron homeostasis are strongly linked to tumorigenesis. Although iron deficiency can hinder cellular growth and metabolic functions, iron overload exerts toxic effects by exacerbating oxidative stress. Through the Fenton reaction, excess iron reacts with hydrogen peroxide (H₂O₂) to produce hydroxyl radicals, highly reactive species that inflict damage on lipids, proteins, and DNA, ultimately initiating ferroptosis(Jiang et al., 2021). Ferroptosis exerts an impact on all facets of cancer cell biology and tumor progression(Bu and Wang, 2024). The mechanisms underlying ferroptosis primarily revolve around three core pathways: iron metabolism and the production of ROS, lipid metabolism, and the system Xc⁻-GSH-GPX4 pathway(Jiang et al., 2021).

The core mechanisms of this process are outlined in Fig. 1, which begins with transferrin-mediated iron uptake via TFR1. Inside the cell, STEAP3 reduces Fe³⁺ to Fe²⁺, which is then transported into the labile iron pool (LIP) by DMT1. Iron storage is managed by ferritin, whose degradation—triggered by its interaction with NCOA4—releases iron back into the LIP(Zheng and Conrad, 2020). Excess Fe²⁺ in the LIP drives the Fenton reaction, generating highly reactive hydroxyl radicals (·OH) that initiate widespread lipid peroxidation, disrupting cellular redox balance and leading to ferroptosis(Jiang et al., 2021). The primary substrates for this peroxidation are polyunsaturated fatty acid phospholipids (PUFA-PLs). Their synthesis begins with acetyl-CoA carboxylase (ACC) producing malonyl-CoA. Subsequently, ACSL4 and LPCAT3 act sequentially to esterify free PUFAs (e.g., arachidonic acid) into membrane phosphatidylethanolamine (PE), forming PUFA-PLs such as AA-PE. These phospholipids are then oxidized by iron-dependent lipoxygenases (LOX) or ·OH into lipid hydroperoxides (e.g., PUFA-PL-OOH), which are the terminal executors of ferroptosis(Li and Li, 2020; Liang et al., 2022). Conversely, monounsaturated fatty acids (MUFAs) can incorporate into membranes and competitively inhibit this peroxidation, thereby suppressing ferroptosis(Pope and Dixon, 2023).

**Fig. E1 Mechanisms underlying ferroptosis.**
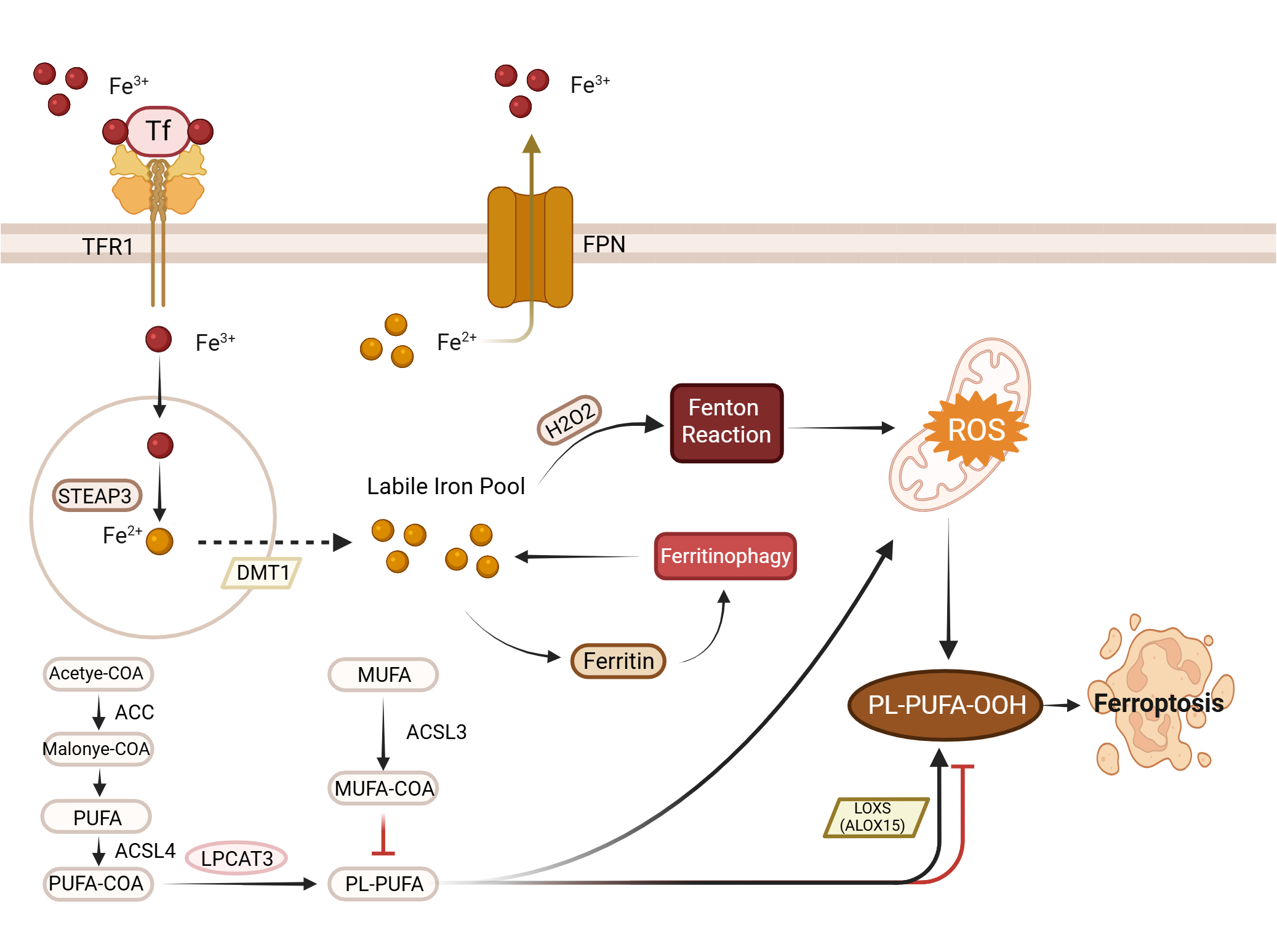


### 2. Specific mechanisms of cuproptosis

Copper is an indispensable trace element in biological systems that functions as a critical enzymatic cofactor, primarily enabling electron transfer reactions in diverse metabolic processes. However, excessive copper accumulation disrupts cellular metabolism and can induce cell death. It is worth noting that serum copper concentrations in cancer patients are markedly higher than those in healthy individuals(Xie et al., 2023).

Cellular copper homeostasis is maintained through coordinated interactions among multiple proteins. As illustrated in Fig. 2, copper uptake is primarily mediated by copper transporter 1 (CTR1/SLC31A1), while mitochondrial copper transport requires cytochrome c oxidase copper chaperone 17 (COX17). Cytosolic and mitochondrial intermembrane copper ions are sequestered by GSH and metallothionein or incorporated into copper chaperones. FDX1 facilitates the reduction of Cu²⁺ to Cu⁺, then Cu⁺ engage with LIAS and subsequently enhance the lipoylation of key metabolic enzymes such as dihydrolipoamide S-acetyltransferase (DLAT). The interaction between Cu⁺ and these lipoylated proteins triggers the formation of toxic oligomers, ultimately leading to cell death. SOD1 acts as a pivotal suppressor of cuproptosis by utilizing copper ions for antioxidant defense(Schmidt et al., 2018).

Copper plays a pivotal role in oncogenesis by modulating key signaling pathways. In murine models of hepatocellular carcinoma, copper overload induces ROS-mediated oxidative stress and activates the TNF-R1 pathway(Lu et al., 2022). Transcriptomic analysis of samples from patients with Neuroblastoma and Brain neoplasm reveal that copper upregulates PD-L1 in tumor cells, promoting immune evasion. Conversely, copper chelators suppress STAT3/EGFR phosphorylation, accelerate PD-L1 degradation via ubiquitination, and enhance tumor infiltration by cytotoxic T lymphocytes (CTLs) and natural killer (NK) cells, thereby attenuating tumor growth and improving survival(Voli et al., 2020).

Under conditions of copper ions overload, Cu²⁺ specifically interacts with lipoylated DLAT, a critical enzyme in the TCA cycle. This copper binding can induce aberrant aggregation of lipoylated proteins and destabilization of Fe-S cluster proteins. These events collectively initiate a proteotoxic stress response that culminates in cell death(Cobine et al., 2021; Chen et al., 2022). Mitochondrial lipoylation represents a critical lysine post-translational modification that facilitates TCA cycle entry by modulating key metabolic enzymes, including: pyruvate dehydrogenase complex component DBT (dihydrolipoyllysine-residue acetyltransferase), glycine cleavage system H protein (GCSH), dihydrolipoamide succinyltransferase (DLST), and DLAT(Tang et al., 2022a). Copper directly coordinates with DLAT, inducing its disulfide-mediated oligomerization through lipid-associated aggregation. As an essential constituent of the pyruvate dehydrogenase (PDH) complex, DLAT mediates the rate-limiting decarboxylation of pyruvate to generate acetyl-CoA, the primary substrate for TCA cycle initiation. FDX1 and lipoic acid synthase (LIAS) function as upstream regulators of DLAT lipoylation. Beyond its role in modulating lipoylated protein function, FDX1 exhibits reductase activity that converts Cu²⁺ to the more redox-active Cu⁺. This reduced copper species directly interacts with lipoylated TCA cycle enzymes, promoting the formation of cytotoxic copper-protein aggregates(Tsvetkov et al., 2022). Elesclomol, a copper ionophore, binds to Cu2+ outside the cell to form a complex (Elesclomol - Cu). This complex enters the cell via membrane transport and releases Cu2+ inside the cell. The released Cu2+ preferentially accumulates in mitochondria. Meanwhile, elesclomol can interact with the mitochondrial enzyme FDX1 to activate mitochondria, induce the production of ROS and Cu+, and thereby kill cancer cells(Tsvetkov et al.,
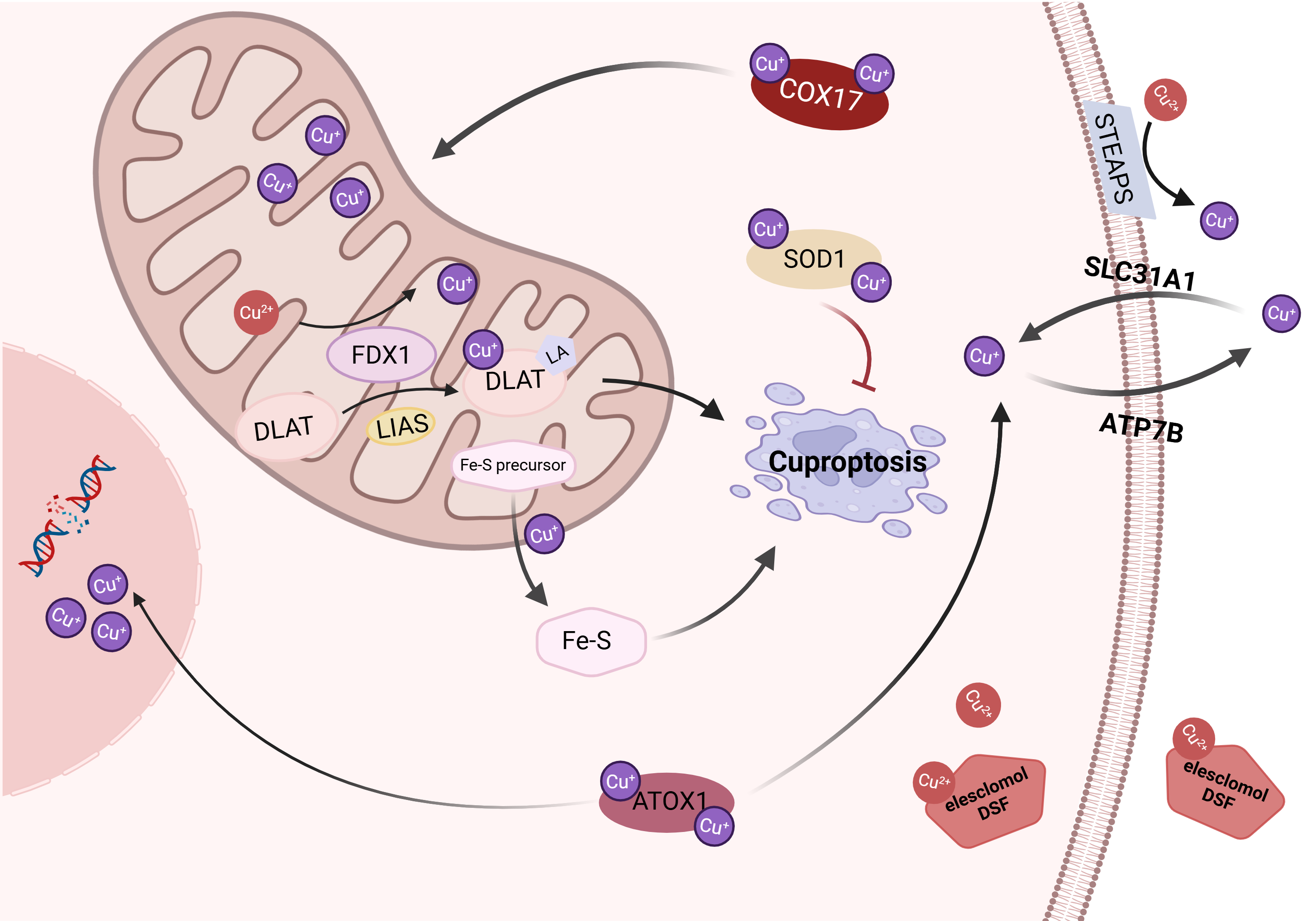
2019).

**Fig. E2 Mechanisms underlying cuproptosis.**

### 3. Mitochondrial metabolism

Previous studies have shown that blocking the TCA cycle or the loss of glutamine can alleviate ferroptosis induced by cystine depletion or the ferroptosis inhibitor erastin(Sabharwal and Schumacker, 2014). In addition, the activity of the mitochondrial electron transport chain (ETC) can also influence the mitochondrial membrane potential, the accumulation of lipid peroxidation products, and the sensitivity of cancer cells to ferroptosis(Gao et al., 2019). For instance, both focal adhesion kinase (FAK) and COX7A1 can enhance the activity of the mitochondrial TCA cycle, thereby enhancing the vulnerability of non-small cell lung cancer (NSCLC) cells to ferroptosis induced by cysteine deprivation(Liu et al., 2020; Feng et al., 2022). Meanwhile, recent research indicates that inhibiting cystathionine β-synthase (CBS) and protein tyrosine phosphatase mitochondrial 1 (PTPMT1) can increase the sensitivity of different tumor cells to cystine deprivation-induced ferroptosis by targeting the TCA cycle(Liu et al., 2024a; Li et al., 2025). The mitochondria in OSCC stem cells feature a fragmented mitochondrial network, and dynamin-related protein 1 (DRP1) is essential for maintaining its spherical shape. DRP1 knockdown-induced mitochondrial dynamics imbalance suppresses the stemness of OSCC cells, increases the level of α-ketoglutarate, and enhances glutaminolysis by upregulating the expression of glutamine transporter ASCT2 to drive the TCA cycle, thereby triggering ferroptosis in OSCC cells(Wang et al., 2024b). Within mitochondria, a central role in combating oxidative reactions is played by DHODH(Zhang et al., 2022a). In ESCC, the overexpression of DHODH leads to an increase in tumor cell proliferation, which can be partially rescued by ferroptosis inhibitors(Shi et al., 2023).

Inhibitors of respiratory chain complexes I/III (rotenone, antimycin A) and mitochondrial pyruvate transporters (UK5099) can significantly inhibit cuproptosis(Tang et al., 2022a). Meantime, multiple risk - scoring models for predicting the prognosis of HNSCC based on cuproptosis - related genes all include members of genes related to the mitochondrial oxidative stress pathway, TCA cycle, and mitochondrial ribosomes. This suggests that mitochondrial function may also play a key role in the regulatory mechanism of cuproptosis(Tang et al., 2022b; Zhang et al., 2022b; Wu et al., 2023b). Notably, mutations in the mitochondrial ribosomal protein MRPS7 impair respiratory chain function, while its overexpression correlates with poor clinical outcomes in HNSCC patients(Menezes et al., 2015). Transcriptomic profiling indicated that DLAT expression was lower in HNSCC compared to adjacent non-tumor tissues and showed a positive correlation with the infiltration of B cells, dendritic cells (DC), Tregs, and CD8+ T cells in HPV+ HNSCC. Meanwhile, both FDX1 and LIAS showed marked downregulation in malignant tissues, indicating suppression of mitochondrial cuproptosis pathways in tumor cells(Jiang et al., 2023; Yang et al., 2023). The latest research has confirmed that low expression of LIAS is significantly positively correlated with better clinical prognosis in ESCC(Wu et al., 2023b).

The flux of the TCA cycle can directly modulate cellular susceptibility to both ferroptosis and cuproptosis. Specifically, molecules such as glutamine (Glu), cytochrome c oxidase subunit 7A1 (COX7A1), and focal adhesion kinase (FAK) accelerate the TCA cycle, thereby promoting both ferroptosis and cuproptosis. In contrast, cystathionine beta-synthase (CBS), protein tyrosine phosphatase mitochondrial 1 (PTPMT1), and kirsten rat sarcoma viral oncogene homolog (KRAS) have the opposite effect, suppressing the cycle. Future efforts should focus on developing mitochondrial-targeted agents that can concurrently induce ferroptosis and cuproptosis, thereby advancing HNSCC treatment.

### 4. GSH metabolism

As the core inhibitory pathway, the Xc⁻-GSH-GPX4 system guards against ferroptosis by continuously detoxifying lipid peroxides.The system Xc⁻ is a heterodimeric membrane transport complex composed of two subunits: solute carrier family 3 member 2 (SLC3A2, CD98hc) and solute carrier family 7 member 11 (SLC7A11, xCT), which are co-expressed and functionally coupled on the plasma membrane surface. This system mediates the efflux of intracellular glutamate and the influx of cystine, reducing cystine to cysteine in the cytoplasm(Ye et al., 2022). As a rate-limiting precursor for GSH synthesis, cysteine can be metabolically degraded to yield pyruvate (PYR) and α-ketoglutarate (α-KG), and PYR is further converted to acetyl coenzyme A (acetyl-CoA). Glutamic acid, glycine, and cysteine are catalyzed by relevant enzymes to synthesize GSH. In the regulation of ferroptosis, GSH, as a key reducing agent and an essential cofactor of GPX4, effectively inhibits the lipid peroxidation reaction by degrading small - molecule peroxides(Lei et al., 2022).

Studies have demonstrated that SLC7A11 overexpression suppresses ferroptosis and consequently promotes lymph node metastasis in ESCC tissues(Feng et al., 2021). Similarly, in HNSCC, GPX4 expression shows a significant correlation with poor patient prognosis. Experimental evidence reveals that GPX4 knockdown reduces tumor cell viability, an effect that can be reversed by ferrostatin-1 pretreatment, confirming GPX4's role as a ferroptosis inhibitor(Lee et al., 2017). Furthermore, multiple microRNAs, including miR-26a-5p, miR-34c-3p, and miR-125b-5p, regulate ferroptosis in HNSCC by targeting SLC7A11 to inhibit system Xc⁻ transport activity, thereby suppressing tumor cell growth(Sun et al., 2022; Liang and Wu, 2023; Yu et al., 2023). Notably, miR-125b-5p upregulation is associated with concurrent SLC1A5 downregulation, which modulates the tumor immune microenvironment and induces ferroptosis, ultimately improving clinical outcomes in HNSCC patients(Liu et al., 2024b). Mechanistically, CirpDe3b binds miR-516b-5p and recruits the HNRNPK/SLC7A11 complex to suppress ferroptosis, consequently promoting ESCC progression(Zhou et al., 2024). Similarly, CircDK8 interacts with miR-615-5p to upregulate SLC7A11 expression while simultaneously reducing ROS, malondialdehyde (MDA), and iron levels. This pathway elevates intracellular GSH and GPX4 concentrations, ultimately inhibiting ferroptosis and enhancing migratory and invasive capacities in OSCC cells(Sun et al., 2024).

Emerging evidence demonstrates that the ferroptosis inducers sorafenib or erastin can potentiate cuproptosis in malignant cells by inhibiting FDX1 degradation and intracellular GSH synthesis. Exogenous supplementation of GSH can significantly reverse the sensitizing effect of ferroptosis inducers on cuproptosis(Chung et al., 2023; Wang et al., 2024a). Recently, several studies have achieved favorable therapeutic outcomes by synergistically enhancing Disulfiram/Copper (DSF/Cu) - induced ferroptosis and cuproptosis in tumor cells through GSH depletion via inhibiting the Xc⁻ - GSH - GPX4 pathway(Li et al., 2024a; Zhang et al., 2024b).

GSH metabolism is the "cross-regulatory hub" of ferroptosis and cuproptosis, and its level can determine the sensitivity of cells to the two death pathways. Compounds such as sorafenib, erastin, and buthionine sulfoximine (BSO) induce ferroptosis and cuproptosis by inhibiting GSH synthesis, while G6PD and p21 can promote this process. However the effect of p53 on GSH synthesis is bidirectional. Although current research on the mechanism of GSH metabolism in regulating cuproptosis in HNSCC is still limited, the above evidence suggests that enhancing cuproptosis sensitivity by inhibiting GSH synthesis may offer a novel therapeutic strategy for HNSCC.

### 5. Autophagy

In ferroptosis, autophagy functions by interfering with various pathways to degrade anti-ferroptosis factors (such as ferritin, lipid droplets, and GPX4). For instance, NCOA4-mediated ferritinophagy facilitates iron accumulation during ferroptosis, whereas RAB7A-mediated lipophagy enhances lipid peroxidation in this identical process(Zhou et al., 2020). Recently, it has been discovered that inducing the autophagic degradation of the iron transporter SLC40A1 can promote ferroptosis and restore the drug sensitivity of cancer cells resistant to ferroptosis(Li et al., 2021a). Inhibiting the expression of autophagy-related genes or transporter receptors can effectively halt the ferroptosis process(Zhao et al., 2025). Emerging evidence demonstrates that ferroptosis activation upregulates lysosome-associated membrane protein type 2A (LAMP2A) expression and triggers chaperone-mediated autophagy (CMA) through an HSP90-dependent pathway. This process facilitates GPX4 degradation, ultimately exacerbating ferroptosis(Wu et al., 2019). Notably, creatine kinase B (CKB) exerts a protective effect by phosphorylating GPX4, which inhibits HSC70-GPX4 binding and consequently blocks CMA-mediated GPX4 degradation. This regulatory mechanism significantly attenuates ferroptosis and promotes tumor progression(Wu et al., 2023a). Autophagy also clears damaged mitochondria through mitophagy, maintains redox balance, and blocks the continuous generation of ROS. This process stands as one of the core pathways governing cellular sensitivity to ferroptosis(Lu et al., 2023). In summary, autophagy exerts a multifaceted and essential influence on ferroptosis through its regulation of iron homeostasis, lipid peroxidation dynamics, antioxidant systems, and organelle functions. Its role can be bidirectional: under certain conditions, autophagy may promote ferroptosis, while in other cases, it may protect cells from ferroptosis by removing damaged cellular components.

In HNSCC xenograft models, pharmacological blockade of glutamine metabolism induces rapid accumulation of polyunsaturated fatty acids (PUFAs) through the autophagy-dependent nutrient sensing pathway, thereby potentiating ferroptosis(Allevato et al., 2024). Mechanistically, protein kinase C iota type (PKCι/ιota) enhances GPX4 protein stability by inhibiting the USP14-mediated autolysosomal degradation pathway, thereby conferring ferroptosis resistance in ESCC cells(Tao et al., 2024). Ubiquitin-specific protease 2 (USP2) promotes ferritin degradation by stabilizing NCOA4, releasing free Fe²⁺ to induce ferroptosis in ESCC cells(Song et al., 2024). The BCL2 family protein BNIP3 promotes mitophagy and consequently sensitizes OSCC cells to cisplatin treatment(Shaw et al., 2020). A recent study on OSCC, showed that acute cadmium ion (Cd²⁺) exposure exerts selective toxicity on OSCC cells derived from non-smokers, while OSCC cells from smokers exhibit resistance due to overexpression of the heavy metal detoxification protein MT2A; Mechanistically, Cd²⁺ competes with iron to induce early iron depletion, driving NCOA4-mediated ferritinophagy, which further increases the LIP, ROS and lipid peroxidation(Petriaggi et al., 2025). Meanwhile, using autophagy inducers and ferroptosis inducers in combination can effectively suppress the proliferation and migration of OSCC(Wang et al., 2023; Zhang et al., 2024a). These findings collectively demonstrate the strong interplay between autophagy and ferroptosis in HNSCC. Notably, most FRGs associated with prognosis in HNSCC participate in in autophagy(Antonelli et al., 2024). For instance, key regulators of phagophore formation and maturation—ATG5, MAP1LC3A, and BECN1—function as autophagy-related biomarkers with prognostic significance in OSCC(Denton and Kumar, 2019). The autophagy-related transcription factor DDIT4 is upregulated in OSCC and is associated with advanced TNM stage, high tumor mutation burden (TMB), as well as poor prognosis characterized by low immune scores and infiltration. It can render cancer cells sensitive to autophagy by inhibiting the mTOR signaling pathway(Zhang et al., 2023b, 2023a). As a prognostic biomarker for HNSCC, the overexpression of CISD2 is significantly associated with tumor drug resistance (Li et al., 2024b). The NEET protein (NAF-1) encoded by this gene can induce cellular autophagy when its function is inhibited. The mechanism may activates ferritinophagy, which subsequently results in a rise in intracellular labile iron levels(Kim et al., 2018).

Dysregulation of copper homeostasis has been demonstrated to simultaneously regulate cuproptosis, ferroptosis, and autophagy activation pathways(Xue et al., 2023a). FDX1, a core gene in cuproptosis, exhibits a positive correlation in expression with autophagy marker genes such as ATG5, ATG12, and BECN-1, suggesting a potential interplay between FDX1-mediated cuproptosis and autophagy(Lu et al., 2022). FDX1 downregulation triggers mitophagy and PI3K/AKT pathway activation, driving tumor progression via ROS accumulation(Chung et al., 2023). Furthermore, copper ions promote oxidative stress, mitochondrial impairment, and DNA damage, initiating apoptosis and autophagy that culminate in cell death(Li et al., 2021b). Research indicates that elevated copper levels activate transcription factor EB (TFEB), enhancing expression of autophagy-related proteins (ATG5, SQSTM1, and MAP1LC3) while modulating the AMPK-mTOR pathway to promote autophagic activity(Guo et al., 2022; Xue et al., 2023a). Copper overload additionally triggers mitophagy via the PINK1/Parkin signaling cascade(Yang et al., 2021). Interestingly, in PDAC models, inhibition of the copper transporter SLC31A1 decreases intracellular copper concentrations yet paradoxically stimulates autophagy(Yu et al., 2019). Currently, the regulatory role of intracellular copper content in autophagy remains controversial. Notably, elevated intracellular copper levels promote GPX4 aggregation by directly binding to the protein. This is followed by the autophagic degradation of GPX4, which ultimately triggers ferroptosis(Xue et al., 2023b). DDX11-AS1, a pivotal lncRNA, is closely linked to the regulation of autophagy and cuproptosis. The PAC-lncRNAs risk model constructed based on DDX11-AS1 demonstrates significant prognostic predictive value and precise clinical stratification capabilities in HNSCC patients(Xiao et al., 2024). Further investigations have demonstrated that the high expression of DDX11-AS1 in ESCC tissues and cells is significantly associated with unfavorable patient prognosis. The underlying mechanism may involve the activation of the Wnt/β-catenin signaling pathway through targeting miR-30d-5p(Guo et al., 2021). The anticancer effect of disulfiram (DSF) in HNSCC is Cu-dependent and mediated through the formation of ROS. Studies have shown that DSF/Cu increases the expression of Beclin-1 and LC3BI/II, which are essential for autophagosome formation, while inhibiting Akt signaling involved in autophagy. These findings suggest that DSF/Cu may induce downregulation of the Akt signaling pathway to promote autophagy, thereby mediating cell death(Park et al., 2018).

## Reference

Allevato, M. M., Trinh, S., Koshizuka, K., Nachmanson, D., Nguyen, T.-T. C., Yokoyama, Y., et al. (2024). A genome-wide CRISPR screen reveals that antagonism of glutamine metabolism sensitizes head and neck squamous cell carcinoma to ferroptotic cell death. *Cancer Letters* 598, 217089. doi: 10.1016/j.canlet.2024.217089

Antonelli, A., Battaglia, A. M., Sacco, A., Petriaggi, L., Giorgio, E., Barone, S., et al. (2024). Ferroptosis and oral squamous cell carcinoma: connecting the dots to move forward. *Front. Oral. Health* 5, 1461022. doi: 10.3389/froh.2024.1461022

Bu, X., and Wang, L. (2024). Iron metabolism and the tumor microenvironment: A new perspective on cancer intervention and therapy (Review). *Int J Mol Med* 55, 39. doi: 10.3892/ijmm.2024.5480

Chen, L., Min, J., and Wang, F. (2022). Copper homeostasis and cuproptosis in health and disease. *Sig Transduct Target Ther* 7, 378. doi: 10.1038/s41392-022-01229-y

Chung, C., Lin, C., Chen, C., Hsueh, C., Chang, Y., Wang, C., et al. (2023). Ferroptosis Signature Shapes the Immune Profiles to Enhance the Response to Immune Checkpoint Inhibitors in Head and Neck Cancer. *Advanced Science* 10, 2204514. doi: 10.1002/advs.202204514

Cobine, P. A., Moore, S. A., and Leary, S. C. (2021). Getting out what you put in: Copper in mitochondria and its impacts on human disease. *Biochimica et Biophysica Acta (BBA) - Molecular Cell Research* 1868, 118867. doi: 10.1016/j.bbamcr.2020.118867

Denton, D., and Kumar, S. (2019). Autophagy-dependent cell death. *Cell Death Differ* 26, 605–616. doi: 10.1038/s41418-018-0252-y

Feng, L., Zhao, K., Sun, L., Yin, X., Zhang, J., Liu, C., et al. (2021). SLC7A11 regulated by NRF2 modulates esophageal squamous cell carcinoma radiosensitivity by inhibiting ferroptosis. *J Transl Med* 19, 367. doi: 10.1186/s12967-021-03042-7

Feng, Y., Xu, J., Shi, M., Liu, R., Zhao, L., Chen, X., et al. (2022). COX7A1 enhances the sensitivity of human NSCLC cells to cystine deprivation-induced ferroptosis via regulating mitochondrial metabolism. *Cell Death Dis* 13, 988. doi: 10.1038/s41419-022-05430-3

Gao, M., Yi, J., Zhu, J., Minikes, A. M., Monian, P., Thompson, C. B., et al. (2019). Role of Mitochondria in Ferroptosis. *Molecular Cell* 73, 354-363.e3. doi: 10.1016/j.molcel.2018.10.042

Guo, H., Ouyang, Y., Yin, H., Cui, H., Deng, H., Liu, H., et al. (2022). Induction of autophagy via the ROS-dependent AMPK-mTOR pathway protects copper-induced spermatogenesis disorder. *Redox Biology* 49, 102227. doi: 10.1016/j.redox.2021.102227

Guo, Y., Sun, P., Guo, W., Yin, Q., Han, J., Sheng, S., et al. (2021). LncRNA DDX11 antisense RNA 1 promotes EMT process of esophageal squamous cell carcinoma by sponging miR-30d-5p to regulate SNAI1/ZEB2 expression and Wnt/β-catenin pathway. *Bioengineered* 12, 11425–11440. doi: 10.1080/21655979.2021.2008759

Jiang, X., Ke, J., Jia, L., An, X., Ma, H., Li, Z., et al. (2023). A novel cuproptosis-related gene signature of prognosis and immune microenvironment in head and neck squamous cell carcinoma cancer. *J Cancer Res Clin Oncol* 149, 203–218. doi: 10.1007/s00432-022-04471-7

Jiang, X., Stockwell, B. R., and Conrad, M. (2021). Ferroptosis: mechanisms, biology and role in disease. *Nat Rev Mol Cell Biol* 22, 266–282. doi: 10.1038/s41580-020-00324-8

Kim, E. H., Shin, D., Lee, J., Jung, A. R., and Roh, J.-L. (2018). CISD2 inhibition overcomes resistance to sulfasalazine-induced ferroptotic cell death in head and neck cancer. *Cancer Letters* 432, 180–190. doi: 10.1016/j.canlet.2018.06.018

Lee, J. R., Roh, J.-L., Lee, S. M., Park, Y., Cho, K.-J., Choi, S.-H., et al. (2017). Overexpression of glutathione peroxidase 1 predicts poor prognosis in oral squamous cell carcinoma. *J Cancer Res Clin Oncol* 143, 2257–2265. doi: 10.1007/s00432-017-2466-7

Lei, G., Zhuang, L., and Gan, B. (2022). Targeting ferroptosis as a vulnerability in cancer. *Nat Rev Cancer* 22, 381–396. doi: 10.1038/s41568-022-00459-0

Li, H., Li, Y., Yu, Y., Ren, X., Yang, C., Jin, W., et al. (2024a). GSH exhaustion via inhibition of xCT-GSH-GPX4 pathway synergistically enhanced DSF/Cu-induced cuproptosis in myelodysplastic syndromes. *Free Radical Biology and Medicine* 222, 130–148. doi: 10.1016/j.freeradbiomed.2024.06.006

Li, J., Liu, J., Xu, Y., Wu, R., Chen, X., Song, X., et al. (2021a). Tumor heterogeneity in autophagy-dependent ferroptosis. *Autophagy* 17, 3361–3374. doi: 10.1080/15548627.2021.1872241

Li, M., Wang, Y., Li, X., Xu, J., Yan, L., Tang, S., et al. (2025). Pharmacological targeting of the mitochondrial phosphatase PTPMT1 sensitizes hepatocellular carcinoma to ferroptosis. *Cell Death Dis* 16, 257. doi: 10.1038/s41419-025-07581-5

Li, Y., Chen, H., Liao, J., Chen, K., Javed, M. T., Qiao, N., et al. (2021b). Long-term copper exposure promotes apoptosis and autophagy by inducing oxidative stress in pig testis. *Environ Sci Pollut Res* 28, 55140–55153. doi: 10.1007/s11356-021-14853-y

Li, Z., Wang, Q., Huang, X., Fu, R., Wen, X., and Zhang, L. (2024b). Multi‐omics analysis reveals that ferroptosis‐related gene CISD2 is a prognostic biomarker of head and neck squamous cell carcinoma. *The Journal of Gene Medicine* 26, e3580. doi: 10.1002/jgm.3580

Liang, B., and Wu, Y. (2023). Hsa-miR-26a-5p improves OSCC sensitivity to ferroptosis by inhibiting SLC7A11. *Archives of Oral Biology* 156, 105807. doi: 10.1016/j.archoralbio.2023.105807

Liu, J., Lu, X., Zeng, S., Fu, R., Wang, X., Luo, L., et al. (2024a). ATF3-CBS signaling axis coordinates ferroptosis and tumorigenesis in colorectal cancer. *Redox Biology* 71, 103118. doi: 10.1016/j.redox.2024.103118

Liu, P., Wu, D., Duan, J., Xiao, H., Zhou, Y., Zhao, L., et al. (2020). NRF2 regulates the sensitivity of human NSCLC cells to cystine deprivation-induced ferroptosis via FOCAD-FAK signaling pathway. *Redox Biology* 37, 101702. doi: 10.1016/j.redox.2020.101702

Liu, Y.-C., Liu, S.-Y., Lin, Y.-C., Liu, C.-J., Chang, K.-W., and Lin, S.-C. (2024b). The disruption of NEAT1-miR-125b-5p-SLC1A5 cascade defines the oncogenicity and differential immune profile in head and neck squamous cell carcinoma. *Cell Death Discov.* 10, 392. doi: 10.1038/s41420-024-02158-1

Lu, H., Zhou, L., Zhang, B., Xie, Y., Yang, H., and Wang, Z. (2022). Cuproptosis key gene FDX1 is a prognostic biomarker and associated with immune infiltration in glioma. *Front. Med.* 9, 939776. doi: 10.3389/fmed.2022.939776

Lu, Y., Li, Z., Zhang, S., Zhang, T., Liu, Y., and Zhang, L. (2023). Cellular mitophagy: Mechanism, roles in diseases and small molecule pharmacological regulation. *Theranostics* 13, 736–766. doi: 10.7150/thno.79876

Menezes, M. J., Guo, Y., Zhang, J., Riley, L. G., Cooper, S. T., Thorburn, D. R., et al. (2015). Mutation in mitochondrial ribosomal protein S7 (MRPS7) causes congenital sensorineural deafness, progressive hepatic and renal failure and lactic acidemia. *Hum. Mol. Genet.* 24, 2297–2307. doi: 10.1093/hmg/ddu747

Park, Y. M., Go, Y. Y., Shin, S. H., Cho, J.-G., Woo, J.-S., and Song, J.-J. (2018). Anti-cancer effects of disulfiram in head and neck squamous cell carcinoma via autophagic cell death. *PLoS ONE* 13, e0203069. doi: 10.1371/journal.pone.0203069

Petriaggi, L., Giorgio, E., Bulotta, S., Antonelli, A., Bonacci, S., Frisina, M., et al. (2025). Acute Exposure to Cadmium Triggers NCOA4-Mediated Ferritinophagy and Ferroptosis in Never-Smokers Oral Cancer Cells. *Int. J. Biol. Sci.* 21, 4131–4152. doi: 10.7150/ijbs.111228

Pope, L. E., and Dixon, S. J. (2023). Regulation of ferroptosis by lipid metabolism. *Trends in Cell Biology* 33, 1077–1087. doi: 10.1016/j.tcb.2023.05.003

Sabharwal, S. S., and Schumacker, P. T. (2014). Mitochondrial ROS in cancer: initiators, amplifiers or an Achilles’ heel? *Nat Rev Cancer* 14, 709–721. doi: 10.1038/nrc3803

Schmidt, K., Ralle, M., Schaffer, T., Jayakanthan, S., Bari, B., Muchenditsi, A., et al. (2018). ATP7A and ATP7B copper transporters have distinct functions in the regulation of neuronal dopamine-β-hydroxylase. *Journal of Biological Chemistry* 293, 20085–20098. doi: 10.1074/jbc.RA118.004889

Shaw, J. J. P., Boyer, T. L., Venner, E., Beck, P. J., Slamowitz, T., Caste, T., et al. (2020). Inhibition of Lysosomal Function Mitigates Protective Mitophagy and Augments Ceramide Nanoliposome–Induced Cell Death in Head and Neck Squamous Cell Carcinoma. *Mol. Cancer Ther.* 19, 2621–2633. doi: 10.1158/1535-7163.MCT-20-0182

Shi, Z.-Z., Jin, X., Li, W.-T., Tao, H., Song, S.-J., Fan, Z.-W., et al. (2023). Dihydroorotate dehydrogenase promotes cell proliferation and suppresses cell death in esophageal squamous cell carcinoma and colorectal carcinoma. *Transl Cancer Res* 12, 2294–2307. doi: 10.21037/tcr-23-136

Song, J., Zhang, J., Shi, Y., Gao, Q., Chen, H., Ding, X., et al. (2024). Hypoxia inhibits ferritinophagy-mediated ferroptosis in esophageal squamous cell carcinoma via the USP2-NCOA4 axis. *Oncogene* 43, 2000–2014. doi: 10.1038/s41388-024-03050-z

Sun, K., Gao, L., Li, S., Zheng, J., Zhu, Z., Zhi, K., et al. (2024). Circ-CDK8 regulates SLC7A11-mediated ferroptosis by inhibiting miR-615-5p to promote progression in oral squamous cell carcinomas. *Front. Pharmacol.* 15, 1432520. doi: 10.3389/fphar.2024.1432520

Sun, K., Ren, W., Li, S., Zheng, J., Huang, Y., Zhi, K., et al. (2022). MiR-34c-3p upregulates erastin-induced ferroptosis to inhibit proliferation in oral squamous cell carcinomas by targeting SLC7A11. *Pathology - Research and Practice* 231, 153778. doi: 10.1016/j.prp.2022.153778

Tang, D., Chen, X., and Kroemer, G. (2022a). Cuproptosis: a copper-triggered modality of mitochondrial cell death. *Cell Res* 32, 417–418. doi: 10.1038/s41422-022-00653-7

Tang, S., Zhao, L., Wu, X.-B., Wang, Z., Cai, L.-Y., Pan, D., et al. (2022b). Identification of a Novel Cuproptosis-Related Gene Signature for Prognostic Implication in Head and Neck Squamous Carcinomas. *Cancers* 14, 3986. doi: 10.3390/cancers14163986

Tao, H., Song, S.-J., Fan, Z.-W., Li, W.-T., Jin, X., Jiang, W., et al. (2024). PKCiota Inhibits the Ferroptosis of Esophageal Cancer Cells via Suppressing USP14-Mediated Autophagic Degradation of GPX4. *Antioxidants* 13, 114. doi: 10.3390/antiox13010114

Tsvetkov, P., Coy, S., Petrova, B., Dreishpoon, M., Verma, A., Abdusamad, M., et al. (2022). Copper induces cell death by targeting lipoylated TCA cycle proteins. *Science* 375, 1254–1261. doi: 10.1126/science.abf0529

Tsvetkov, P., Detappe, A., Cai, K., Keys, H. R., Brune, Z., Ying, W., et al. (2019). Mitochondrial metabolism promotes adaptation to proteotoxic stress. *Nat Chem Biol* 15, 681–689. doi: 10.1038/s41589-019-0291-9

Voli, F., Valli, E., Lerra, L., Kimpton, K., Saletta, F., Giorgi, F. M., et al. (2020). Intratumoral Copper Modulates PD-L1 Expression and Influences Tumor Immune Evasion. *Cancer Res.* 80, 4129–4144. doi: 10.1158/0008-5472.CAN-20-0471

Wang, L., Wang, C., Li, X., Tao, Z., Zhu, W., Su, Y., et al. (2023). Melatonin and erastin emerge synergistic anti-tumor effects on oral squamous cell carcinoma by inducing apoptosis, ferroptosis, and inhibiting autophagy through promoting ROS. *Cell Mol Biol Lett* 28, 36. doi: 10.1186/s11658-023-00449-6

Wang, Y., Chen, Y., Zhang, J., Yang, Y., Fleishman, J. S., Wang, Y., et al. (2024a). Cuproptosis: A novel therapeutic target for overcoming cancer drug resistance. *Drug Resistance Updates* 72, 101018. doi: 10.1016/j.drup.2023.101018

Wang, Z., Tang, S., Cai, L., Wang, Q., Pan, D., Dong, Y., et al. (2024b). DRP1 inhibition-mediated mitochondrial elongation abolishes cancer stemness, enhances glutaminolysis, and drives ferroptosis in oral squamous cell carcinoma. *Br J Cancer* 130, 1744–1757. doi: 10.1038/s41416-024-02670-2

Wu, K., Yan, M., Liu, T., Wang, Z., Duan, Y., Xia, Y., et al. (2023a). Creatine kinase B suppresses ferroptosis by phosphorylating GPX4 through a moonlighting function. *Nat Cell Biol* 25, 714–725. doi: 10.1038/s41556-023-01133-9

Wu, Z., Geng, Y., Lu, X., Shi, Y., Wu, G., Zhang, M., et al. (2019). Chaperone-mediated autophagy is involved in the execution of ferroptosis. *Proc. Natl. Acad. Sci. U.S.A.* 116, 2996–3005. doi: 10.1073/pnas.1819728116

Wu, Z., Huang, Z., Zhou, X., Gao, C., Peng, Z., Zheng, X., et al. (2023b). Comprehensive analysis of cuproptosis genes and cuproptosis-related genes as prognosis factors in esophageal squamous cell carcinoma. *Genomics* 115, 110732. doi: 10.1016/j.ygeno.2023.110732

Xiao, Y., Li, Q., and Li, Y. (2024). Exploring the role of long noncoding RNAs in autophagy and cuproptosis processes via immune pathways in head and neck squamous carcinoma: A systematic review of the literature. *Medicine (Baltimore)* 103, e39335. doi: 10.1097/MD.0000000000039335

Xie, J., Yang, Y., Gao, Y., and He, J. (2023). Cuproptosis: mechanisms and links with cancers. *Mol Cancer* 22, 46. doi: 10.1186/s12943-023-01732-y

Xue, Q., Kang, R., Klionsky, D. J., Tang, D., Liu, J., and Chen, X. (2023a). Copper metabolism in cell death and autophagy. *Autophagy* 19, 2175–2195. doi: 10.1080/15548627.2023.2200554

Xue, Q., Yan, D., Chen, X., Li, X., Kang, R., Klionsky, D. J., et al. (2023b). Copper-dependent autophagic degradation of GPX4 drives ferroptosis. *Autophagy* 19, 1982–1996. doi: 10.1080/15548627.2023.2165323

Yang, F., Liao, J., Yu, W., Qiao, N., Guo, J., Han, Q., et al. (2021). Exposure to copper induces mitochondria-mediated apoptosis by inhibiting mitophagy and the PINK1/parkin pathway in chicken (Gallus gallus) livers. *Journal of Hazardous Materials* 408, 124888. doi: 10.1016/j.jhazmat.2020.124888

Yang, Q., Zeng, S., and Liu, W. (2023). Roles of cuproptosis-related gene DLAT in various cancers: a bioinformatic analysis and preliminary verification on pro-survival autophagy. *PeerJ* 11, e15019. doi: 10.7717/peerj.15019

Ye, Y., Chen, A., Li, L., Liang, Q., Wang, S., Dong, Q., et al. (2022). Repression of the antiporter SLC7A11/glutathione/glutathione peroxidase 4 axis drives ferroptosis of vascular smooth muscle cells to facilitate vascular calcification. *Kidney International* 102, 1259–1275. doi: 10.1016/j.kint.2022.07.034

Yu, Y., MohamedAl‐Sharani, H., and Zhang, B. (2023). EZH2‐mediated SLC7A11 upregulation via miR‐125b‐5p represses ferroptosis of TSCC. *Oral Diseases* 29, 880–891. doi: 10.1111/odi.14040

Yu, Z., Zhou, R., Zhao, Y., Pan, Y., Liang, H., Zhang, J., et al. (2019). Blockage of SLC31A1‐dependent copper absorption increases pancreatic cancer cell autophagy to resist cell death. *Cell Proliferation* 52, e12568. doi: 10.1111/cpr.12568

Zhang, L., Li, Z., Ma, X., Yang, W., Hao, Y., Zhang, L., et al. (2024a). Combination treatment with ferroptosis and autophagy inducers signiﬁcantly inhibit the proliferation and migration of oral squamous cell carcinoma. *Biochemical and Biophysical Research Communications* 709, 149842. doi: 10.1016/j.bbrc.2024.149842

Zhang, P., Zhou, C., Ren, X., Jing, Q., Gao, Y., Yang, C., et al. (2024b). Inhibiting the compensatory elevation of xCT collaborates with disulfiram/copper-induced GSH consumption for cascade ferroptosis and cuproptosis. *Redox Biology* 69, 103007. doi: 10.1016/j.redox.2023.103007

Zhang, S., Kang, L., Dai, X., Chen, J., Chen, Z., Wang, M., et al. (2022a). Manganese induces tumor cell ferroptosis through type-I IFN dependent inhibition of mitochondrial dihydroorotate dehydrogenase. *Free Radical Biology and Medicine* 193, 202–212. doi: 10.1016/j.freeradbiomed.2022.10.004

Zhang, S., Zhang, L., Lu, H., Yao, Y., Liu, X., and Hou, J. (2022b). A cuproptosis and copper metabolism–related gene prognostic index for head and neck squamous cell carcinoma. *Front. Oncol.* 12, 955336. doi: 10.3389/fonc.2022.955336

Zhang, Y., Liu, L., Hou, X., Zhang, Z., Zhou, X., and Gao, W. (2023a). Role of Autophagy Mediated by AMPK/DDiT4/mTOR Axis in HT22 Cells Under Oxygen and Glucose Deprivation/Reoxygenation. *ACS Omega* 8, 9221–9229. doi: 10.1021/acsomega.2c07280

Zhang, Z., Zhu, H., Zhao, C., Liu, D., Luo, J., Ying, Y., et al. (2023b). DDIT4 promotes malignancy of head and neck squamous cell carcinoma. *Molecular Carcinogenesis* 62, 332–347. doi: 10.1002/mc.23489

Zhao, P., Yin, S., Qiu, Y., Sun, C., and Yu, H. (2025). Ferroptosis and pyroptosis are connected through autophagy: a new perspective of overcoming drug resistance. *Mol Cancer* 24, 23. doi: 10.1186/s12943-024-02217-2

Zhou, B., Liu, J., Kang, R., Klionsky, D. J., Kroemer, G., and Tang, D. (2020). Ferroptosis is a type of autophagy-dependent cell death. *Seminars in Cancer Biology* 66, 89–100. doi: 10.1016/j.semcancer.2019.03.002

Zhou, P., Wu, Z., Zhang, Q., Wang, L., Zhang, W., and Han, X. (2024). A novel link between circPDE3B and ferroptosis in esophageal squamous cell carcinoma progression. *Genomics* 116, 110761. doi: 10.1016/j.ygeno.2023.110761
