# Supplementary material for: Ferroptosis and cuproptosis in head and neck squamous cell carcinoma: interconnected mechanisms and therapeutic implications
Source: Front Pharmacol. 2026 Feb 13;17:1694895. doi: 10.3389/fphar.2026.1694895 (PMC12946670; doi:10.3389/fphar.2026.1694895)
Supplement: Supplementary file 2 [file Supplementaryfile2.docx]

**Supplementary material 2**

### The Specific Mechanisms of p53 and NRF2 in the Crosstalk between Ferroptosis and Cuproptosis

#### 1. p53

The tumor suppressor p53 exhibits context-dependent regulation of ferroptosis, functioning as either an inhibitor or promoter(Tarangelo et al., 2018). p53 induces ferroptosis by suppressing the expression of SLC7A11 or directly acting on spermidine/spermine N1-acetyltransferase 1 (SAT1) and mitochondrial glutaminase 2 (GLS2). It downregulates SLC7A11 expression either by interacting with ubiquitin-specific protease 7 (USP7) or directly binding to the SLC7A11 promoter. This suppression inhibits system Xc⁻ activity, ultimately inducing ferroptosis in tumor cells. Concurrently, high p53 expression can also directly target SAT1 and GLS2 to regulate glutamine metabolism, indirectly triggering ferroptosis(Tarangelo et al., 2018; Kang et al., 2019). However, under specific conditions, p53 can also inhibit the ferroptosis process by activating cyclin-dependent kinase inhibitor 1A (CDKN1A)/p21 or inhibiting dipeptidyl peptidase 4 (DPP4)(Xie et al., 2017).

p53 promotes the TCA cycle and oxidative phosphorylation by inhibiting glycolytic pathways and enhancing mitochondrial metabolic activity. Moreover, p53 modulates both iron-sulfur cluster biogenesis and GSH synthesis—critical regulators of cuproptosis—implicating its potential involvement in cuproptosis regulation(Matoba et al., 2006; Xiong et al., 2023). Research demonstrates that during glutamine deprivation, p53 upregulates SLC1A3 expression - encoding an aspartate/glutamate transporter - to sustain aspartate metabolic flux and support TCA cycle function(Tajan et al., 2018). p53 has been found to modulate the expression of multiple genes involved in Fe-S cluster biogenesis, such as HSPA9, FDXR, ISCU, and FXN, thereby participating in Fe-S cluster assembly. HSPA9 is a chaperone protein for Fe-S cluster assembly. Studies have demonstrated that the tumor-suppressive micropeptide miPEP133, encoded by the primary miR-34a transcript and activated by p53, specifically interacts with the mitochondrial chaperone protein HSPA9. This interaction induces functional inhibition of HSPA9, subsequently decreasing mitochondrial membrane potential, reducing mitochondrial content, and potentially disrupting Fe-S cluster biosynthesis(Kang et al., 2020). Additionally, p53 reduces NADPH production by inhibiting either malic enzyme or the pentose phosphate pathway, thereby diminishing GSH levels(Jiang et al., 2011, 2013). However, p53 also enhances GSH biosynthesis by activating various metabolic genes (including TIGAR(Bensaad et al., 2006), GLS2(Hu et al., 2010), SESN1/2, and CDKN1A(Budanov et al., 2004)) to protect cells against oxidative toxicity, thereby suppressing ferroptosis and cuproptosis. The regulation of iron/copper homeostasis by p53 is collectively influenced by stress intensity, cell type, and metabolic context, reflecting its conditional dependence and bidirectional regulatory properties in the control of programmed cell death (PCD). p53 also plays an important role in autophagy regulation; activated p53 triggers autophagic cell death by activating AMPK signaling(Puissant et al., 2010) or DRAM expression(Hasei et al., 2013). Under conditions of glucose limitation, AMPK-mediated phosphorylation activates p53, establishing a positive feedback mechanism that couples p53 activity with cellular glucose metabolism(Jones et al., 2005). Additionally, p53 promotes autophagy activation by inhibiting the mTOR signaling pathway. Clinical studies demonstrate that genetic variations in the mTOR pathway occur in approximately 30% of OSCC cases, and this pathway significantly regulates malignant phenotypes of OSCC cells, including proliferation, invasion, migration, angiogenesis, and metabolic reprogramming. Currently, mTOR-targeted inhibitors have shown promising therapeutic potential in OSCC clinical trials(Su et al., 2022).

In summary, p53 orchestrates cell fate decisions in a context-dependent manner through a multifaceted network that regulates lipid metabolism, glutathione homeostasis, iron-sulfur cluster assembly, and autophagy.

#### 2. NRF2

Under cellular stress, nuclear translocation of NRF2 enables its binding to antioxidant response elements (AREs) in promoter regions, initiating transcription of downstream antioxidant genes. This regulatory mechanism coordinates diverse metabolic processes encompassing protein homeostasis, xenobiotic detoxification, iron regulation, as well as carbohydrate and lipid metabolism. The association between NRF2 and ferroptosis has recently attracted widespread attention(Jiang et al., 2024). NRF2 is significantly overexpressed in various solid tumors, including lung cancer, liver cancer, and HNSCC. Current research about the regulatory role of NRF2 in ferroptosis within HNSCC has primarily focused on the Xc⁻-GSH-GPX4 antioxidant system. Overall, NRF2 activation confers resistance to ferroptosis in cancer cells(Sun et al., 2016; Kerins and Ooi, 2018). High NRF2 expression induces elevated SLC7A11 levels, which enhances system Xc⁻ function and increases GPX4 expression, ultimately suppressing ROS and lipid peroxidation. Conversely, NRF2 knockout reduces GSH levels in HNSCC cells(Chen et al., 2009; Chung et al., 2023). The endogenous NRF2 inhibitor Kelch-like ECH-associated protein 1 (KEAP1) demonstrates significantly elevated expression in HNSCC tissues compared to normal mucosa(Shin et al., 2018). microRNAs miR-153 and miR-125b have been shown to enhance ferroptotic sensitivity in HNSCC through NRF2 downregulation(Yang and Gu, 2024). Emerging evidence indicates that KRAS targeting promotes ferroptosis in hepatocellular carcinoma (HCC) via coordinated modulation of both the TCA cycle and the NRF2-SLC7A11-GPX4 axis(Zhang et al., 2025).

Recently, a growing body of research has demonstrated the important role of NRF2 in regulating cuproptosis, with its mechanisms involving the regulation of ion metabolism, intermediate metabolism, GSH metabolism, and antioxidant stress responses(Tang et al., 2024). Copper ion overload can activate the NRF2 signaling pathway to upregulate the expression of metallothionein-1/2 (MT-1/MT-2). MT proteins can chelate Cu ions transported into cells by SLC31A1 and inhibit the trafficking of ATP7A from the trans-Golgi complex to the plasma membrane, thereby suppressing cuproptosis(Gudekar et al., 2020). Notably, as a primary copper ion chelator, GSH can exert a buffering effect before being bound by MT proteins. The expression of glutamate-cysteine ligase regulatory subunit (GCLM) and catalytic subunit (GCLC)—the rate-limiting enzymes for GSH synthesis—is also directly regulated by the NRF2 signaling pathway(Liu et al., 2024). Researchers such as Cheng confirmed that circSpna2 can competitively bind to KEAP1 through a molecular sponge effect, thereby relieving KEAP1-mediated inhibition of NRF2. This upregulates NRF2 expression levels, significantly enhances the expression of the copper transporter ATP7B, and ultimately promotes intracellular copper ion efflux, effectively alleviating copper ion accumulation in depression after traumatic brain injury in a mouse model(Du et al., 2024). In another study, inhibition of the NRF2 signaling pathway combined with upregulation of FDX1 expression activated endoplasmic reticulum stress and induced cuproptosis, thereby suppressing cervical cancer cells invasion and migration(Zhang et al., 2024).

## Reference

Bensaad, K., Tsuruta, A., Selak, M. A., Vidal, M. N. C., Nakano, K., Bartrons, R., et al. (2006). TIGAR, a p53-Inducible Regulator of Glycolysis and Apoptosis. *Cell* 126, 107–120. doi: 10.1016/j.cell.2006.05.036

Budanov, A. V., Sablina, A. A., Feinstein, E., Koonin, E. V., and Chumakov, P. M. (2004). Regeneration of Peroxiredoxins by p53-Regulated Sestrins, Homologs of Bacterial AhpD. *Science* 304, 596–600. doi: 10.1126/science.1095569

Chen, W., Sun, Z., Wang, X.-J., Jiang, T., Huang, Z., Fang, D., et al. (2009). Direct Interaction between Nrf2 and p21Cip1/WAF1 Upregulates the Nrf2-Mediated Antioxidant Response. *Molecular Cell* 34, 663–673. doi: 10.1016/j.molcel.2009.04.029

Chung, C., Lin, C., Chen, C., Hsueh, C., Chang, Y., Wang, C., et al. (2023). Ferroptosis Signature Shapes the Immune Profiles to Enhance the Response to Immune Checkpoint Inhibitors in Head and Neck Cancer. *Advanced Science* 10, 2204514. doi: 10.1002/advs.202204514

Du, M., Fu, J., Zhang, J., Zhu, Z., Huang, X., Tan, W., et al. (2024). CircSpna2 attenuates cuproptosis by mediating ubiquitin ligase Keap1 to regulate the Nrf2‐Atp7b signalling axis in depression after traumatic brain injury in a mouse model. *Clinical &amp; Translational Med* 14, e70100. doi: 10.1002/ctm2.70100

Gudekar, N., Shanbhag, V., Wang, Y., Ralle, M., Weisman, G. A., and Petris, M. J. (2020). Metallothioneins regulate ATP7A trafficking and control cell viability during copper deficiency and excess. *Sci Rep* 10, 7856. doi: 10.1038/s41598-020-64521-3

Hasei, J., Sasaki, T., Tazawa, H., Osaki, S., Yamakawa, Y., Kunisada, T., et al. (2013). Dual Programmed Cell Death Pathways Induced by p53 Transactivation Overcome Resistance to Oncolytic Adenovirus in Human Osteosarcoma Cells. *Mol. Cancer Ther.* 12, 314–325. doi: 10.1158/1535-7163.MCT-12-0869

Hu, W., Zhang, C., Wu, R., Sun, Y., Levine, A., and Feng, Z. (2010). Glutaminase 2, a novel p53 target gene regulating energy metabolism and antioxidant function. *Proc. Natl. Acad. Sci. U.S.A.* 107, 7455–7460. doi: 10.1073/pnas.1001006107

Jiang, P., Du, W., Mancuso, A., Wellen, K. E., and Yang, X. (2013). Reciprocal regulation of p53 and malic enzymes modulates metabolism and senescence. *Nature* 493, 689–693. doi: 10.1038/nature11776

Jiang, P., Du, W., Wang, X., Mancuso, A., Gao, X., Wu, M., et al. (2011). p53 regulates biosynthesis through direct inactivation of glucose-6-phosphate dehydrogenase. *Nat Cell Biol* 13, 310–316. doi: 10.1038/ncb2172

Jiang, X., Yu, M., Wang, W., Zhu, L., Wang, X., Jin, H., et al. (2024). The regulation and function of Nrf2 signaling in ferroptosis-activated cancer therapy. *Acta Pharmacol Sin* 45, 2229–2240. doi: 10.1038/s41401-024-01336-2

Jones, R. G., Plas, D. R., Kubek, S., Buzzai, M., Mu, J., Xu, Y., et al. (2005). AMP-Activated Protein Kinase Induces a p53-Dependent Metabolic Checkpoint. *Molecular Cell* 18, 283–293. doi: 10.1016/j.molcel.2005.03.027

Kang, M., Tang, B., Li, J., Zhou, Z., Liu, K., Wang, R., et al. (2020). Identification of miPEP133 as a novel tumor-suppressor microprotein encoded by miR-34a pri-miRNA. *Mol Cancer* 19, 143. doi: 10.1186/s12943-020-01248-9

Kang, R., Kroemer, G., and Tang, D. (2019). The tumor suppressor protein p53 and the ferroptosis network. *Free Radical Biology and Medicine* 133, 162–168. doi: 10.1016/j.freeradbiomed.2018.05.074

Kerins, M. J., and Ooi, A. (2018). The Roles of NRF2 in Modulating Cellular Iron Homeostasis. *Antioxidants & Redox Signaling* 29, 1756–1773. doi: 10.1089/ars.2017.7176

Liu, J., Tang, H., Chen, F., Li, C., Xie, Y., Kang, R., et al. (2024). NFE2L2 and SLC25A39 drive cuproptosis resistance through GSH metabolism. *Sci Rep* 14, 29579. doi: 10.1038/s41598-024-81317-x

Matoba, S., Kang, J.-G., Patino, W. D., Wragg, A., Boehm, M., Gavrilova, O., et al. (2006). p53 Regulates Mitochondrial Respiration. *Science* 312, 1650–1653. doi: 10.1126/science.1126863

Puissant, A., Robert, G., Fenouille, N., Luciano, F., Cassuto, J.-P., Raynaud, S., et al. (2010). Resveratrol Promotes Autophagic Cell Death in Chronic Myelogenous Leukemia Cells via JNK-Mediated p62/SQSTM1 Expression and AMPK Activation. *Cancer Res.* 70, 1042–1052. doi: 10.1158/0008-5472.CAN-09-3537

Shin, D., Kim, E. H., Lee, J., and Roh, J.-L. (2018). Nrf2 inhibition reverses resistance to GPX4 inhibitor-induced ferroptosis in head and neck cancer. *Free Radical Biology and Medicine* 129, 454–462. doi: 10.1016/j.freeradbiomed.2018.10.426

Su, Y.-C., Lee, W.-C., Wang, C.-C., Yeh, S.-A., Chen, W.-H., and Chen, P.-J. (2022). Targeting PI3K/AKT/mTOR Signaling Pathway as a Radiosensitization in Head and Neck Squamous Cell Carcinomas. *IJMS* 23, 15749. doi: 10.3390/ijms232415749

Sun, X., Ou, Z., Chen, R., Niu, X., Chen, D., Kang, R., et al. (2016). Activation of the p62‐Keap1‐NRF2 pathway protects against ferroptosis in hepatocellular carcinoma cells. *Hepatology* 63, 173–184. doi: 10.1002/hep.28251

Tajan, M., Hock, A. K., Blagih, J., Robertson, N. A., Labuschagne, C. F., Kruiswijk, F., et al. (2018). A Role for p53 in the Adaptation to Glutamine Starvation through the Expression of SLC1A3. *Cell Metabolism* 28, 721-736.e6. doi: 10.1016/j.cmet.2018.07.005

Tang, D., Kroemer, G., and Kang, R. (2024). Targeting cuproplasia and cuproptosis in cancer. *Nat Rev Clin Oncol* 21, 370–388. doi: 10.1038/s41571-024-00876-0

Tarangelo, A., Magtanong, L., Bieging-Rolett, K. T., Li, Y., Ye, J., Attardi, L. D., et al. (2018). p53 Suppresses Metabolic Stress-Induced Ferroptosis in Cancer Cells. *Cell Reports* 22, 569–575. doi: 10.1016/j.celrep.2017.12.077

Xie, Y., Zhu, S., Song, X., Sun, X., Fan, Y., Liu, J., et al. (2017). The Tumor Suppressor p53 Limits Ferroptosis by Blocking DPP4 Activity. *Cell Reports* 20, 1692–1704. doi: 10.1016/j.celrep.2017.07.055

Xiong, C., Ling, H., Hao, Q., and Zhou, X. (2023). Cuproptosis: p53-regulated metabolic cell death? *Cell Death Differ* 30, 876–884. doi: 10.1038/s41418-023-01125-0

Yang, J., and Gu, Z. (2024). Ferroptosis in head and neck squamous cell carcinoma: from pathogenesis to treatment. *Front. Pharmacol.* 15, 1283465. doi: 10.3389/fphar.2024.1283465

Zhang, J., Liu, Z., Zhao, W., Li, C., Liu, F., and Wang, J. (2025). Targeting KRAS Sensitizes Ferroptosis by Coordinately Regulating the TCA Cycle and Nrf2‐SLC7A11‐GPX4 Signaling in Hepatocellular Carcinoma. *Smart Medicine* 4, e70005. doi: 10.1002/smmd.70005

Zhang, M., Shi, M., Yu, Y., Ou, R., Ge, R., and Duan, P. (2024). Curcuminoid PBPD induces cuproptosis and endoplasmic reticulum stress in cervical cancer via the Notch1/RBP‐J/NRF2/FDX1 pathway. *Molecular Carcinogenesis* 63, 1449–1466. doi: 10.1002/mc.23735
